# Extrinsically Conductive Nanomaterials for Cardiac Tissue Engineering Applications

**DOI:** 10.3390/mi12080914

**Published:** 2021-07-31

**Authors:** Arsalan Ul Haq, Felicia Carotenuto, Paolo Di Nardo, Roberto Francini, Paolo Prosposito, Francesca Pescosolido, Fabio De Matteis

**Affiliations:** 1 Department of Clinical Sciences and Translational Medicine, University of Rome Tor Vergata, Via Montpellier 1, 00133 Rome, Italy; carotenuto@med.uniroma2.it (F.C.); dinardo@uniroma2.it (P.D.N.); francesca.pescosolido@alumni.uniroma2.eu (F.P.); 2 CIMER, Centre for Regenerative Medicine, University of Rome Tor Vergata, Via Montpellier 1, 00133 Rome, Italy; francini@roma2.infn.it (R.F.); prosposito@roma2.infn.it (P.P.); Fabio.dematteis@uniroma2.it (F.D.M.); 3 L.L. Levshin Institute of Cluster Oncology, I.M. Sechenov First Moscow State Medical University, 119992 Moscow, Russia; 4 Industrial Engineering Department, University of Rome Tor Vergata, Via del Politecnico 1, 00133 Rome, Italy

**Keywords:** cardiac tissue engineering, conductive nanomaterials, myocardial infarction, cardiovascular disease, ischemic tissue repair

## Abstract

Myocardial infarction (MI) is the consequence of coronary artery thrombosis resulting in ischemia and necrosis of the myocardium. As a result, billions of contractile cardiomyocytes are lost with poor innate regeneration capability. This degenerated tissue is replaced by collagen-rich fibrotic scar tissue as the usual body response to quickly repair the injury. The non-conductive nature of this tissue results in arrhythmias and asynchronous beating leading to total heart failure in the long run due to ventricular remodelling. Traditional pharmacological and assistive device approaches have failed to meet the utmost need for tissue regeneration to repair MI injuries. Engineered heart tissues (EHTs) seem promising alternatives, but their non-conductive nature could not resolve problems such as arrhythmias and asynchronous beating for long term in-vivo applications. The ability of nanotechnology to mimic the nano-bioarchitecture of the extracellular matrix and the potential of cardiac tissue engineering to engineer heart-like tissues makes it a unique combination to develop conductive constructs. Biomaterials blended with conductive nanomaterials could yield conductive constructs (referred to as extrinsically conductive). These cell-laden conductive constructs can alleviate cardiac functions when implanted in-vivo. A succinct review of the most promising applications of nanomaterials in cardiac tissue engineering to repair MI injuries is presented with a focus on extrinsically conductive nanomaterials.

## 1. Introduction

Cardiovascular diseases (CVDs) are the leading cause of death globally with 523 million active cases as of 2019 and claimed 18.6 million lives the same year [1]. According to the World Heart Federation, the global cost to cope with CVDs could rise to an astounding 1044 billion USD by 2030 from 863 billion USD in 2010 [2]. In Europe alone, CVDs claim 3.9 million lives each year and costs 210 billion EUR while ischemic heart disease costs about 59 billion EUR each year to the EU economy [3]. Myocardial infarction is one of the clusters of numerous other CVDs. Obstruction of the coronary artery due to blood clots/plaques results in myocardial tissue ischemia and necrosis which consequently leads to infarction [4]. This results in the loss of billions of contractile cardiomyocytes (CMs) with poor regeneration capability [5,6]. The injured tissue is replaced with the collagen-rich fibrotic scar tissue that induces arrhythmias due to its inability to conduct electric currents [7]. The current pharmacological approach administering drugs such as β-blockers, renin-angiotensin system inhibitors, β_2_ adrenergic receptor agonist clenbuterol etc. are known to reverse ventricular remodelling by activating anti-inflammatory pathways and reducing apoptosis in ischemic rodent models [8,9,10,11,12]. Furthermore, the ventricular assistive device approach can also provide a short term solution to the failing heart but these strategies fail to meet the utmost need for tissue regeneration to mend the ischemic insult [13,14,15]. Therefore, the long term solution remains the heart transplant so far but the shortage of organ donors and the possibility of immune rejection makes this approach elusive [16]. 

**Figure 1:**
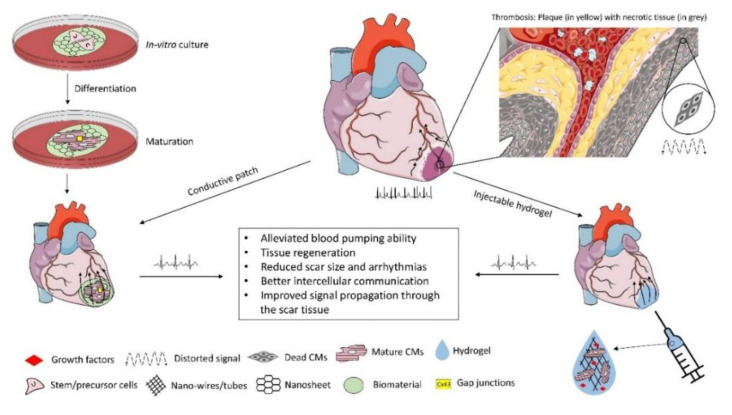
Conductive scaffold-based Cardiac Tissue Engineering. Due to infarction, collagen-rich scar tissue significantly impairs the electrical conductivity of the myocardium. Cell-laden nanomaterial-based conductive constructs can restore the electrical activity of the damaged myocardium with alleviated functions when implanted in-vivo. Some of the figures were adopted from the Servier Medical Art and modified under the CC BY 3.0 license.

Cell-laden structures made of natural or synthetic biomaterials to mimic the extracellular matrix (ECM) can emulate the biomechanical properties of the cardiac tissue providing efficient substitutes to the injured myocardium [17,18,19,20,21,22]. However, when implanted in-vivo the issues related to the arrhythmias and asynchronous beating of the injured myocardium are not resolved due to the non-conductive nature of the conventional engineered tissues. Collagens I and III fibrils are important constituents of the ECM with the diameter ranging from 150–300 nm and 25–100 nm, respectively [23]. The conductive Purkinje fibres in the heart are around 70–80 µm thick [24]. The potential of nanotechnology to mimic these micro/nano constituents, cardiac tissue bio-architecture and conductivity makes it a unique combination with tissue engineering to develop biomimetic conductive constructs in-vitro [25]. Two routes can be followed to develop conductive constructs for tissue engineering applications: (i) using intrinsically conductive organic polymers such as polyaniline, polypyrrole and poly(3,4-ethylenedioxythiophene) [26], (ii) or extrinsically conductive inorganic nanomaterials such as metal/carbon-based nanoparticles, nanotubes, nanowires, nanosheets etc. embedded in the inert biomaterials. Intrinsically conductive polymers are referred to as organic polymers which are doped with either p-type or n-type dopant that creates native polarons or bipolarons within the polymer structure thus imparting conductivity [27]. On the other hand, inorganic nanomaterials embedded in biomaterials induce a different mechanism of conduction thus referred to as extrinsically conductive in this article. Both structures, when seeded with cardiac cells yield heart-like tissues in-vitro which could decrease the resistivity of the fibrotic scar tissue when implanted in-vivo. This in turn would result in better propagation of the electrical signals through it overcoming the issues regarding arrhythmias. The nurturing electrical microenvironment can also enhance the cell-cell communications between the ischemic and seeded cells leading to better proliferation and replacement of the ischemic tissue in the long run. Figure 1 illustrates the conductive nanomaterials-based-cardiac tissue engineering scheme. 

The most promising applications of extrinsically conductive nanomaterials in cardiac tissue engineering to repair MI injuries are succinctly reviewed as follows.

## 2. Cardiac ECM and Post-MI Remodelling

The cardiac extracellular matrix contains various components such as collagen, elastin, laminin, glycoproteins, and glycosaminoglycans. Advances in proteomics revealed that about 90% of the cardiac ECM is composed of 10 major proteins out of which serum albumin, collagens (type I, III and IV), non-fibrillar fibronectin and laminin, elastin, proteoglycans and glycosaminoglycan are the most abundant [28]. Cardiac ECM is divided into two main sections (i) interstitial matrix and (ii) basement membrane. Interstitial matrix is mainly composed of fibrillar collagens (80% type I and >10% type III) which are anchored to the basement membrane composed of non-fibrillar collagen IV, fibronectin, laminin, proteoglycans and glycosaminoglycans [29].

**Figure 2:**
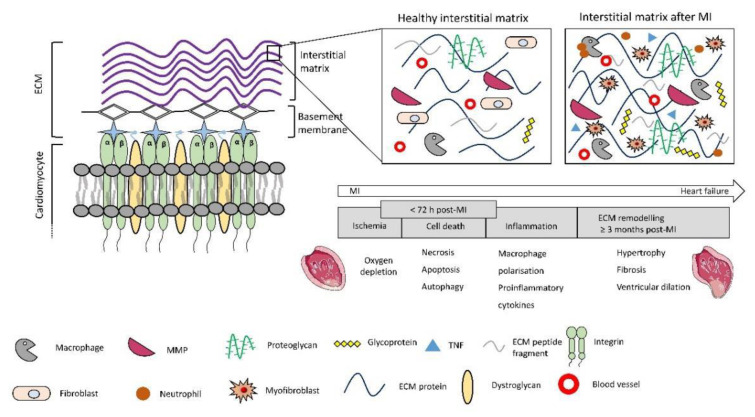
ECM components: Interstitial matrix consists of fibrillar collagen, elastin, proteoglycans and glycoproteins, basement membrane is made of non-fibrillar collagen and laminin. Cardiomyocytes attach to the basement membrane via transmembrane proteins such as integrin and Dystroglycan. The excessive ECM secretion after the infarction leads to tissue hypertrophy and scar formation. The figure of the MI heart was adopted from the Servier Medical Art under the CC BY 3.0 license.

Collagen fibrils which are around 100 nm in diameter provide structural integrity, elasticity to the tissue, and anchor to the basement membranes [30]. Thick elastin fibrils around 0.2 µm in diameter provide elasticity to the tissue while laminin (77 nm long arm, 36 nm short arm) forms the basement membranes [31]. Fibronectin (61 nm length, 2 nm diameter) which is a glycoprotein binds to other ECM components [32]. The nano-size and complicated interlinks between these components impart complex anisotropic properties to the ECM which is difficult to mimic. MI triggers intense tissue repair mechanisms involving ventricular remodelling followed by inflammation and scar formation [33]. The excessive ECM secretion post-MI results in tissue hypertrophy with increased stiffness resulting in scar formation which hampers the electrical conductivity of the myocardium [34,35,36]. Within the first 30 min post-MI, CMs physiology is seriously compromised resulting in irreversible cell death that triggers acute inflammatory pathways [37,38]. Neutrophils and macrophages infiltrating the infarcted site release the inflammatory mediators such as tumour necrosis factors (TNF) and matrix metalloproteinases (MMPs) for ECM degradation and removal of the dead tissue/CMs debris [39]. After day 1, the neutrophils population exceeds that of macrophage’s and peaks by day 3 and after a week both start to decline [40,41,42]. The inflammatory cascades also turn cardiac fibroblasts into their activated state (myofibroblasts) that secrete out excessive ECM as shown in Figure 2. Scar tissue rich in collagen begins to form within five days post-MI to replace the extensive CMs loss [43]. To repair the post-ischemic insult to the myocardium, a construct should mimic the nano-architecture of the cardiac ECM to provide cells with an in-vivo-like microenvironment. Given below are some fabrication techniques that can be used to control structural features of the construct at micro/nano scale. These constructs can then be seeded with cells to develop heart-like tissues in-vitro to repair the MI injuries. 

## 3. Fabrication Techniques to Develop Micro/Nano-Structured Constructs

Pristine/pure nanomaterials are difficult to use for tissue engineering applications due to their poor processability and potential to elicit cytotoxicity in the long run. Therefore, they are usually blended with other biomaterials to yield biocompatible and electroconductive constructs. Traditional fabrication techniques cannot fabricate scaffolds that can mimic the nano-architecture of the ECM. Therefore, micro/nanofabrication techniques such as 3D printing, photolithography, electrospinning etc. are used presently to engineer biomimetic constructs with ECM-like nano-architecture. These cell-laden conductive constructs can be implanted at the infarction site to repair the injured heart explained in detail in Section 5.

### 3.1. Photolithography

The two-photon polymerisation technique is a versatile technique to achieve sub-50 nm resolutions to mimic the tissue bioarchitecture [44]. In this technique, a femtosecond laser pulse is focused on the volume of a photosensitive solution. This pulse then initiates a photo-polymerisation reaction via free radical mechanism by the absorption of two extremely focused photons into a tiny spot in the solution creating nanostructures. For example, a cardiac patch (2 mm × 2 mm × 100 µm) was fabricated using methacrylated gelatin/photo-initiator as bioink and seeded with human induced pluripotent stem cells (hiPSCs) derived CMs, smooth muscle cells and endothelial cells. Just after one day, the cultured cells started to beat synchronously, and after four weeks the cell-laden patch significantly alleviated the cardiac functions in murine MI models when implanted in-vivo [45]. Similarly, direct laser writing (DLW) is also a useful technique to achieve sub-100 nm resolutions [46]. In this technique, a visible/UV laser beam is focused on a photosensitive solution and the continuous movement of the laser along x-y axes makes 2D layers via free radical photo-polymerisation. The downward movement of the stage along the *z*-axis deposits these 2D layers on top of the other to develop a 3D construct. Unlike the two-photon polymerisation technique, it does not produce an extremely focused laser beam, therefore, fine structures below 50 nm cannot be fabricated. Projection micro-stereolithography (PµSL) is another useful technique to fabricate scaffolds within 5–10 µm resolution [47,48]. In this technique, conductive nanomaterials are mixed with photosensitive resins and visible light photo-initiators such as irgacure 819, Eosin Y etc. are used to make a 3D construct. The resin is shined with the impression of the desired shape through photomask making 2D layers and the stage is lowered along the *z*-axis to deposit many layers to make a 3D construct. 

### 3.2. 3D Bioprinting

3D bioprinting has emerged as an outstanding tool to make artificial tissues with resolution ranging from 50–500 µm depending on the targeted tissue/organ [49]. In this technique, conductive nanomaterial, biomaterials, and cells are blended to make bioink which is used to make 3D constructs via a layer-by-layer deposition approach. There are 3 main modes of 3D bioprinting, (i) Inkjet bioprinting, (ii) Pressure-assisted bioprinting, and (iii) Laser-assisted bioprinting. In all these modes the fabrication process is usually divided into six steps. (1) Imaging: the damaged tissue or organ is scanned to make a blueprint for the bioprinter. Several imaging techniques such as X-ray, MRI, CAT scan is used for this purpose. (2) Design approach: several approaches such as biomimicry, tissue self-assembly to develop organs, and the bottom-up assembly of the smaller building blocks of the tissue/organ. (3) Materials selection: material selection is tissue/organ specific. Natural/synthetic biomaterials or decellularised ECM could be used to develop bioink for the printer. (4) Cell selection: cell source could be autologous, allogenic, xenogeneic, or induced pluripotent stem cells. (5) Bioprinting mode: the selected design, material and cell population must integrate with the chosen inkjet, pressure-assisted or laser-assisted mode. (6) Application: some tissues might require some maturation time in the bioreactor before in-vivo applications and some could be used right away [50]. 

In a study, CNTs imparted nano-filament like structure to the bioprinted constructs which led to the improved elongation and attachment of human coronary artery endothelial cells. Cellular proliferation and differentiation led to the formation of lumen-like structures in-vitro after ten days [51]. Bioprinted microporous constructs utilising conductive bioink containing gold nanoparticles (NPs) and CNTs significantly improved various cellular behaviours of the cultured cells with strong cardiac-like phenotype [52,53]. 

### 3.3. Electrospinning

Electrospinning is a useful technique to mimic the fibrous tissue architecture, particularly the ECM. In this technique, a conductive solution is prepared by blending biomaterials with nanomaterials and it is then loaded in the syringe. The potential difference is set up between the syringe and the collector. This difference in potential works as the driving force to protrude the fibres out of the needle and the continuous deposition of these fibres yields nanofibrous membranes. These conductive membranes have shown the potential to drive cardiomyogenic differentiation of various stem cell lines. For example, CNTs and gold NPs incorporated fibrous membranes directed hiPSCs and hMSCs alignment along the fibre lengths giving them sarcomeric morphology and the cells differentiated to cardiomyocyte-like cells with synchronous beating patterns [54,55,56].

### 3.4. Other Techniques

Dip-pen nanolithography is a versatile nano-fabrication technique. In this technique, AFM tip (pen) is dipped in the solution/ink containing molecular units such as collagen, peptides etc. and these units are transferred onto the rigid surface one by one via capillary action. Both soft and hard tissues with sub-50 nm architecture can be printed with this method. The sequential or parallel deposition of multiple compounds would enable mimicking the complex architecture of the ECM with many different components [57]. Micro-contact printing (µCP) is another inexpensive technique to fabricate 2D templates with micropatterns mimicking the striated heart morphology. A PDMS stamp with micropatterns is prepared first and then it is immersed in the conductive ink containing nanomaterials. An impression is stamped on a surface yielding 2D conductive constructs. This groove-ridge topography unidirectionally aligns the cultured cells as observed in the case of embryonic stem cell-derived cardiac progenitor cells which formed a contractile myocardial tissue layer with striated morphology [58]. Table 1 demonstrates fabrication techniques to develop micro/nano-structured constructs. 

## 4. Biological Response of the Cultured Cells to the Conductive Scaffolds

**Figure 3:**
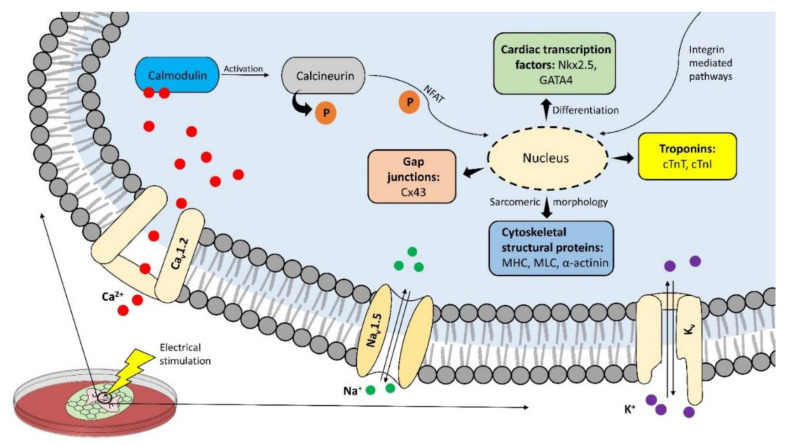
Conductive nano-constructs can trigger intracellular signalling pathways when cultured with stem/precursor cells particularly when stimulated electrically. These pathways then lead to the elevated expression of cardiac-specific genes and transcription factors resulting in the cardiac phenotype of the cultured cells.

Cells cultured on the conductive scaffold behave differently compared to when cultured on the non-conductive scaffold. The spontaneous action potentials fired by the cultured cardiomyocytes can propagate easily throughout the conductive scaffold. The voltage-gated ion channels (Na_v_1.5, Ca_v_1.2, and K_v_) open in response when the excitation potential crosses the resting membrane potential of −90 mV in the case of cardiomyocytes [65], while in the case of stem/precursor cells these channels could be opened by an external electrical stimulation [66]. Due to this, the disturbance in ionic concentration takes place across the plasma membrane resulting in ionic influx and membrane depolarisation. Calcium ions activate the calcium-binding calmodulin protein which in turn activates calcineurin via its dephosphorylation [67]. Intracellular signals leading to cardiac-like phenotype, are then activated by phosphorylated NFAT triggered and integrin-mediated MAPK/ERK pathways [68,69,70]. The expression of cardiac-specific transcription factors (Nkx2.5 and GATA4) indicate the differentiation of the cultured stem/precursor cells to cardiomyocyte-like cells with improved intercellular communication demonstrated by gap junction proteins. Connexin 43 (Cx43) is the principal gap junction protein of the heart which mediates action potential propagation between cells to synchronise cardiac contraction. In addition to this canonical role, it may act as the transcription regulator [71]. GATA4 and Nkx2.5 are cardiac-specific transcription factors that play a fundamental role in myocardial differentiation and cardiac hypertrophy in the early stages of cardiogenesis in a developing embryo while cardiac troponins are expressed in the cardiac muscles of the mammalian and avian species [72,73,74]. The expression of actomyosin structural proteins such as myosin heavy chain (MHC), myosin light chain (MLC) and α-actinin result in the formation of contractile machinery of the cells facilitating their synchronous beating. Improved assembly of intercalated discs (ID) in differentiated cardiomyocyte-like cells could also be observed by the enhanced expression of ID-related N-cadherin, plakophilin, plakoglobin proteins [75,76]. This way, the nanomaterial-based conductive scaffold could direct the differentiation of the cultured stem cells to cardiomyocyte-like cells demonstrating the cardiac phenotype. The cell-scaffold interaction is described schematically in Figure 3 and in detail in the following sections. 

### 4.1. Effect of Conductive Nano-Constructs on Cell Viability and Proliferation

Owing to their excellent electrical conductivity and potential to enhance intercellular coupling effects carbon nanotubes-based conductive scaffolds are quite appealing in tissue engineering and biomedical applications [77,78,79,80,81,82]. For example, carbon nanotubes (CNTs) embedded in polyurethane/chitosan membranes significantly improved the viability of H9c2 cardiomyoblasts after one week of culture [83]. However, the small size of single-wall carbon nanotubes/SWCNTs (1–3 nm diameter) could often pose complications due to plasma membrane penetration which could disrupt the intracellular processes in the long run [84,85,86,87]. To avoid it, multi-wall carbon nanotubes/MWCNTs (10–200 nm diameter) were blended with decellularised pericardial matrix and after one week, cultured HL-1 myocytes demonstrated a high proliferation rate with cardiac phenotype [88,89]. Graphene oxide is less conductive compared to its reduced form. The partially reduced graphene oxide foam demonstrated an electrical conductivity of 112 S/m [90] while the electrical conductivity of the culture medium lies between 1.68 to 1.728 S/m [91]. The low oxygen content of partially reduced graphene attracted the protonated/deprotonated forms of proteins from the culture medium leading to better cell attachment. After ten days, CMs ubiquitously organised themselves throughout the 3D porous foam and started to protrude branches while aggregating at different locations. These branches then merged forming a larger beating syncytium with 65 bpm [90]. This reveals the biocompatibility of pristine graphene oxide substrate without offering any potential cytotoxicity. Carbon nanofibers and gold nanowires/nanorods also directed the cardiac cell alignment along the conductive strands resulting in cardiac phenotype when blended with other biomaterials [92,93]. The high surface area of TiO_2_ nanoparticles incorporated into chitosan increased the cell-matrix interactions. This eventually led to the formation of an interconnected cardiac tissue layer when cultured with CMs [94]. The electrical connectivity between H9c2 cardiomyoblasts and chitosan-selenium NPs film mediated cellular attachment and migration with a high proliferation rate. Cells ended up making a cardiac tissue layer due to this enhanced connectivity [95]. When stimulated (2 ms, 1 Hz, 5 V/cm) for five days, cardiomyocyte viability significantly increased and demonstrated a relatively stronger cardiac phenotype compared to the unstimulated group when cultured on thiol-2-hydroxyethyl methacrylate/Au-NPs hydrogels [96]. Porcine cholecystic derived ECM conjugated with gold NPs provided nurturing microenvironment to the cultured H9c2 cells. Compared to the non-conductive constructs, incorporation of gold NPs significantly increased the cell viability with reduced apoptosis rate 24 h post culture [97]. Table 2 summarises the nurturing effect of nanomaterial-based conductive constructs on cell viability and proliferation. 

### 4.2. Effect of Conductive Nano-Constructs on Cellular Differentiation

Biomaterials blended with extrinsically conductive nanomaterials have been shown to direct the differentiation of the cultured stem cells to cardiomyocyte-like cells (Table 3). Highly directional 1D fullerene whiskers directed the differentiation of C2C12 myoblasts towards myotubes evident from the enhanced expression of MyoD and myogenin. Compared to the random myotube formation on the glass, fullerene whiskers led to the formation of multinucleated myotubes with high fusion and maturation indices after 24 h of culture [98]. When stimulated electrically (8 V, 10 ms, 1 Hz) for two days myotubes demonstrated a strong cardiac phenotype with the expression of MRF4, MHC, myogenin and α-actinin up-regulated several folds [99]. Gold-coated polycaprolactone (PCL) membranes also generated myotubes when cultured with H9c2 cells. Myotubes demonstrated high maturation and fusion indices with 4.6 folds increase in MHC expression compared to when cells were cultured on non-conductive PCL membranes [100]. Likewise, 129/SVE-derived mouse stem cells created embryoid bodies (EBs) when cultured on gelatin methacrylate (GelMA)/CNTs hydrogels. EBs could be thought of as a 3D bundle of stem cells mimicking the early developmental stages of an embryo [101]. When stimulated electrically, EBs started to beat with 1.8 beats/s compared to EBs formed on non-conductive GelMA hydrogels. Several folds increase in the expression of cardiac-specific Nkx2.5 (14.8 folds), cTnT2 (59.6 folds), Tbx5 (3.9 folds), and Myh7 (4.9 folds) genes was observed when CNTs were embedded inside of the embryoid bodies. This shows that CNTs embedded EBs differentiated to cardiomyocyte-like cells with significant contractions when stimulated electrically [102,103]. Fullerene containing culture medium and conductive alginate/fullerenol hydrogels triggered the ERK/MAPK pathways which led to the differentiation of brown adipose-derived stem cells (BADSCs) to cardiomyocyte-like cells. Cells demonstrated twice the Cx43 expression compared to when cultured on non-conductive constructs [104,105]. Likewise, MWCNTs directed the myocardial differentiation of unrestricted somatic stem cells with a high proliferation rate. Cells demonstrated enhanced expression of cardiac troponin I (cTnI), Cx43 and β-MHC with high intracellular activity when stimulated electrically [106].

The significant potential of conductive nano-constructs to mediate myocardial differentiation of adult stem cells to cardiac-like cells gives hope to avoid the use of embryonic stem cells which involve many ethical concerns. In some studies, mesenchymal stem cells and cardiac progenitor cells differentiated to cardiomyocyte-like cells when cultured on reduced graphene oxide (rGO) and gold NPs incorporated constructs. Differentiated cells demonstrated enhanced expression of cardiac-specific genes revealing an in-vitro possibility to engineer heart-like tissues using nanomaterials [107,108,109,110].

### 4.3. Effect of Conductive Nano-Constructs on Cell Morphology

Cells can sense the conductive surface and can adapt their morphology accordingly particularly along the conductive struts as shown in various studies (Table 4). It was observed when neonatal rat CMs aligned themselves along the nanotube lengths when cultured on pristine MWCNTs films. Cells developed heart tissue-like sarcomeric morphology and due to the conductive nature of the nanotubes, cells started to beat with the same frequency as that of the pacemaker [112]. Similarly, the incorporation of gold NPs into laponite loaded myocardial ECM hydrogels led to the formation of mature CMs with elongated sarcomeres and enhanced expression of Cx43, and cTnI compared to when cultured on non-conductive hydrogels [113]. SWCNTs incorporated into collagen substrate led to intercalated discs (IDs) formation when cultured with neonatal rat ventricular myocytes (NRVMs). The presence of nanotubes triggered the β1 integrin-mediated pathways which led to the expression of mechanical and junctional proteins while this effect was not observed in pristine collagen cultured cells [114]. NRVMs directed their growth along the conductive fibres when cultured on Au-NPs loaded PCL/gelatin electrospun membranes. Cells developed synchronous contractions with an eight-folds increase in the contraction amplitude compared to when cultured on non-conductive membranes [115]. Likewise, gold nanorods incorporated into GelMA hydrogels also triggered the β1 integrin-mediated pathways which improved the cell-matrix interactions [116]. Furthermore, rGO and carbon nanofibrous substrates resulted in the spatial cytoskeletal organisation of the cultured CMs with strong cardiac sarcomeric morphology evident from the enhanced expression of Cx43, α-actinin and cardiac troponin [117,118]. An electrical stimulation (10 ms, 1 Hz, 5 V) sent the electrical pulses throughout the conductive fibres of PCL/graphene electrospun membranes which were sensed by the cultured mouse embryonic stem cells derived CMs. In response to this stimulation, cells started to align and elongate themselves along the conductive fibres in the direction of pulse propagation. Eventually, cells displayed sarcomeric morphology evident from up-regulated levels of MHC, cTnT and β-actin compared to when cultured on non-conductive PCL membranes [119].

### 4.4. Effect of Conductive Nano-Constructs on the Electrical Coupling of Cells and Contractility

Pristine carbon nanotubes or when blended with other biomaterials have been shown to substantially enhance the electrical coupling among the cultured cells due to their conductive nature (Table 5). In one of the studies, NRVMs and cardiac fibroblasts developed tight desmosome-like nano-connections when cultured on pristine MWCNTs films [121]. Cells developed rhythmic contractions with high beating frequencies compared to when cultured on non-conductive constructs [122]. The propagation of electrical signals through the nanotube structure significantly enhanced the intercellular coupling with high expression of gap junction protein among cultured CMs and synchronous beating when stimulated electrically [112,123]. However, when cultured on substrates with high nanotube concentrations, CMs developed arrythmias pointing out the potential cytotoxicity of nanotubes if used in higher concentrations [124]. Carbon nanofibers blended with chitosan also led to improved intercellular coupling with a mature contractile phenotype of the cultured neonatal rat CMs after fourteen days. Several folds increase in the expression of Cx43, MHC, troponins, GATA4 and ANF led to the formation of contractile cytoskeletal structure compared to when cells were cultured on non-conductive substrates [125]. Atrial natriuretic factor (ANF) is a hormone secreted out by the cardiac cells mainly in the atrial walls during stretching and it maintains the circulatory homeostasis [126,127]. Electro-mechanical features of the cardiac patch were tuned changing the graphene content to provide cells with an in-vivo-like elastic and electrical microenvironment. EBs generated from embryonic stem cells started to beat after twelve days of culture with twice the beating rate compared to control [128]. Likewise, cardiac cells started to beat when cultured on graphene-based conductive hydrogels with twice the amplitude of intracellular calcium transients [129] and the beating frequency synchronised with the frequency (0.5 Hz) of the applied electrical stimulation. However, at higher frequencies (2–3 Hz) cells developed arrythmias [130]. Gold NPs incorporated in decellularised omental matrix increased the contraction amplitude of the CMs by two-folds with faster calcium transients [131]. Excellent electrical conductivity of gold nanowires incorporated in alginate patch led to 2.4 folds increase in Cx43 expression with highly synchronised calcium transients compared to poor transients in the case of cells cultured on pristine alginate [93]. 

### 4.5. Conductive Nanomaterials as a Vehicle for Gene Delivery

Conductive nanomaterials are a good vehicle to deliver various genes at the infarction site for therapeutic purposes (Table 6). In one of the studies, deoxyribozymes or DNA enzymes (DNAzyme) were delivered to the infarction site after conjugating with gold nanoparticles via intramyocardial injection. Cy5-stained DNAzyme conjugated AuNPs catalytically silenced about 50% of tumour necrosis factor (TNF-α) using primary macrophage as a model. This resulted in significant up-regulation of anti-inflammatory pathways and improved the various functions of the infarcted myocardium [134]. Likewise, thiol modified antago-miR155 conjugated with gold nanoparticles delivered the nucleic acids into macrophages through phagocytosis via tail vein injection in ovariectomised diabetic mice. This in-vivo delivery of antagon-miR155 appreciably increased the anti-inflammatory type 2 macrophage (M2) levels compared to pro-inflammatory type 1 macrophage (M1) levels. This in turn led to reduced apoptosis and restored cardiac functions evident from elevated expression of CD68^+^ cells, and miR155 with improved blood pumping ability [135]. Intraperitoneally injection of circ-Amotl1-PEG conjugated with gold nanoparticles in mice led to improved CMs survival. Circular RNA, circ-Amolt1 physically bound to the PDK1 and AKT1 in-vivo and facilitated cardio-protection against Doxorubicin-induced cardiomyopathy. Doxorubicin is a drug used in chemotherapy and its long-term use can induce cardiomyopathy [136].

Single wall carbon nanotube (SWCNT) was functionalised with siRNA from the Caspase3 gene referred to as F-SWCNT-siCase3. It demonstrated 85% transfection efficiency in-vitro which significantly down-regulated the expression of Caspase3 related RNA and proteins in CMs. This Caspase3 gene silencing led to enhanced cell viability and cardiac functions after intramyocardial injection of F-SWCNT-siCase3 in SD rats. One week post-injection, LV wall thickness increased by 1.42-folds compared to control with decreased infarct size [137]. Similarly, functionalised graphene nanosheet was conjugated with DNA_VEGF_ referred to as fGO_VEGF_ encapsulated inside GelMA hydrogel for localised and controlled release of the gene. Intramyocardial injection of fGO_VEGF_/GelMA complex at the peri-infarct site in rats led to improved angiogenesis with reduced scar size. Two weeks post-injection, fGO_VEGF_/GelMA injected group demonstrated better cardiac performance compared to DNA_VEGF_/GelMA, GelMA and sham groups evident from echocardiography [138].

## 5. In-Vivo Ischemic Tissue Repair

Nanomaterial-based conductive scaffolds can also reduce further ventricular dilation and improve the functions of the infarcted myocardium as observed in animal models. Graphene oxide/silk fibroin hydrogel loaded with cardiac progenitor cells significantly reduced the scar size with improved regeneration of the ventricular wall when injected in-vivo [139]. Four weeks post-injection, the infarcted wall demonstrated intercalated discs formation with twice the thickness when injected with oligo(poly(ethylene glycol) fumarate)/graphene oxide hydrogels [120]. Furthermore, the infarcted heart demonstrated improved blood pumping ability while the scar size reduced by 2.3 folds compared to MI hearts [140]. In-vivo efficacy of cell-laden constructs is determined if it can alleviate the blood pumping ability of the injured heart with improved regeneration of the LV wall. Collagen/carbon nanofibrous conductive constructs led to significant regeneration of the infarcted wall. After four weeks, the regenerated wall demonstrated sarcomeric morphology with high angiogenesis evident from the enhanced expression of α-actinin and CD31, respectively [92]. An appreciable population of brown adipose-derived stem cells survived the hostile MI environment when injected with conductive fullerenol/alginate and poly(N-isopropylacrylamide)/SWCNT hydrogels. Cells differentiated to cardiomyocyte-like cells regenerating the LV wall with 3 folds increase in the wall thickness while scar size reduced by two-folds. Conductive constructs improved the blood pumping ability of the injured myocardium and angiogenesis with thick blood vessels [105,111]. These results justify the in-vivo efficacy of nanomaterials-based conductive scaffolds to regenerate the infarcted myocardium as shown in Table 7.

## 6. Limitations

This review also underlines certain limitations of the nanomaterials for cardiac tissue engineering applications. For example, the preclinical studies shown in Table 7 should not be taken as the next valid step towards clinical studies due to differences in the physiology of humans and animals and various other factors. Moreover, nanomaterial-based cardiac tissue engineering is still in its infancy and a lot of work needs to be done before it could enter clinical trials. 

Despite the substantial effects of the nanomaterials on various cellular behaviours, it is worth mentioning that nanomaterials could pose serious complications such as their potential cytotoxicity and non-biodegradable debris could elicit inflammation at the local tissue site for long-term in-vivo applications. Several risks factors of nanomaterials such as toxicity, carcinogenicity, genotoxicity, immunogenicity, and teratogenicity are often dose dependent. These factors may lead to cancer, dysfunction of the various body systems such as reproductive system, harmful effects on the foetus before birth etc. due to their long-term presence inside the body. Furthermore, a 2020 report released by the Organisation for Economic Co-operation and Development (OECD) determined the three most common upstream key factors of tissue injury following nanomaterial exposure (i) Inflammation, (ii) Oxidative stress, and (iii) Cytotoxicity. This induced tissue injury due to these factors may lead to tissue dysfunction which ultimately results in adverse outcomes such as fibrosis, emphysema, granuloma, and mesothelioma [142]. Biopersistence and biodurability of conductive nanomaterials have also been shown to activate NLRP3 inflammasome on various stem cell lines. This, in turn, caused lysosomal membrane permeabilisation (LMP) and autophagy dysfunction [143]. 

The lack of international standards, regulatory tools, and methods for the risk assessment of nanomaterials is another constraint to use nanomaterials for biomedical and tissue engineering applications as these standards are costly and laborious. Various regional risk assessment frameworks exist to date [144,145] with their own scope, pros and cons and almost all of them lack decision making criteria to use nanomaterials for practical applications except one. This detailed framework developed by the European Centre for Ecotoxicology and Toxicology of Chemicals (ECETOC) is mainly focused on human health hazards of nanomaterials. It uses three tier system to set up decision-making criteria for grouping nanomaterials into four categories and testing them [146]. Even though this decision-making framework is fully elaborated and covers the life cycle of nanomaterials and the biological pathways, its regulatory acceptability is unclear. Furthermore, it mainly focuses on the inhalation hazards of nanomaterials, not all risks. Due to the lack of such standards, it is difficult to launch conductive nanomaterial-based commercial products for cardiac therapy limiting their clinical applications.

The conventional scaffolds fabrication processes such as gas foaming, particulate leaching, solvent casting, freeze-drying, phase separation etc. offer certain limitations to produce conductive scaffold with micro/nano structural features. These limitations usually include-dimensional constraints, limited control over pore geometry and their interconnectivity, use of toxic organic compounds etc. Current fabrication techniques such as 3D printing, photolithography, bioprinting etc. have eliminated most of these constraints but they still lack in mimicking nano-architectural features of cardiac tissue. Furthermore, these techniques particularly lithography sometimes require high operating expertise and are expensive as we tend to go down to nano scale to achieve nano/sub-micro architectural features in a scaffold. One of the biggest limitations of current fabrication techniques is their inability to control scaffold design niches at the molecular scale. At this scale, the biological interaction of cells with the construct is completely different compared to the interactions with the bulk material. These cell-matrix interactions at this scale would mediate cells to develop in-vivo like cardiac phenotype thus developing conductive heart-like tissues in-vitro. 

Finally, PubMed and Google Scholar were used for the literature survey using certain keywords: (“cardiac” OR “tissue” OR “engineering” OR “nanomaterials”) AND (“nanomaterials” OR “conductive” OR “scaffolds” OR “infarction”). Due to the focus of the searching algorithm on these keywords, we may not have been able to include all the relevant studies even after a careful search by trying different combinations of these keywords. 

## 7. Conclusions and Future Perspectives

The possibility that the heart would repair itself after infarction is restricted due to the poor regeneration capability of the CMs. The usual injury repair mechanism of the body kicks in quickly compared to the least probable tissue regeneration mechanism, sealing the injury with scar tissue. Compared to the current pharmacological approaches mitigating only the symptoms of end-stage heart failure, left ventricular assistive devices (LVADs) have shown some potential with a patient surviving as long as 7 years after an LVAD transplant [147]. However, the heart transplant remains the gold standard to date for long-term solutions with a median survival rate of around 12 to 13 years [148]. Cardiac tissue engineering seems a promising alternative when it comes to repairing MI related injuries. Not only it eliminates the issues regarding LVAD device complications but also the paucity of organ donors and the possibility of immune rejection in the case of a heart transplant. With the emerging applications of nanotechnology in tissue engineering and regenerative medicine, conductive heart-like tissues closely mimicking the nano bio-architecture of the cardiac ECM can be engineered in-vitro. 

The preclinical results of these conductive constructs are encouraging but we still need to address certain challenges. Even though nanomaterials can mimic the physicochemical properties of the ECM components it is still difficult to control the construct design niches at the molecular scale. Actually, the translation from the theoretical continuum mechanical framework of soft biological tissues to somewhat practical heart-like tissues is arduous due to the design limitations offered by the current fabrication techniques. Poor understanding of the heart biomechanics is another constraint because of its anisotropic nature. A group tried to unveil the biomechanical properties of the human myocardial tissues. They performed biaxial extension and triaxial shear tests on different myocardial tissue specimens harvested from the donor heart considered unfit for the transplantation due to certain reasons. For the left ventricle wall, the maximum Cauchy stress value varied from 2.6 to 18.9 kPa in the direction of myocardial fibres orientation while it varied from 1.3 to 10.5 kPa perpendicular to the fibres at different stretch levels from λ = 1.05 to λ = 1.15. On the other hand, the maximum shear stress value among six different shear modes was 5.8 ± 1.7 kPa [149]. For a scaffold to mimic the biomechanical properties of the myocardium this study could be a guide. Table 8 narrows down different studies as a guide for the selection of the best possible parameters to design a conductive scaffold/approach to resolve different cardiac-related issues. Gold nanoparticles seem to be the best candidates for gene delivery while carbon-based nanomaterials seem more suitable for tissue engineering applications. 

To conclude, new fabrication techniques which would allow us to control the construct design niches at the molecular scale is a dire need in tissue engineering and regenerative medicine fields. With this approach conductive constructs closely mimicking the heart bio-architecture could be developed. However, for now, on this long avenue of cardiac tissue engineering, the short-term goal is to restore the electrophysiological features of the ischemic tissue using the conductive scaffold-based approach to address the issues regarding arrythmias and asynchronous beating of the injured myocardium. The long-term goal is the lab-grown hearts successfully underwent the trial phases to clinically address the problem. 

## Figures and Tables

**Table 1:** Techniques to fabricate micro/nano-structured constructs.

	Photolithography	3D Bioprinting	Electrospinning	Dip-Pen Nanolithography	Micro-Contact Printing
Resolution	50 nm–10 µm	50–500 µm	100 nm–150 µm	20–30 nm	35 nm–1 µm
Pros	Precise structural control	A wide variety of biomaterials, nanomaterials and cells can be incorporated	Suitable to mimic fibrous ECM structure	Precise control over the complex architecture	Finely detailed structures, printing on uneven surfaces
Cons	Expensive, potential cytotoxic compounds, time-consuming	Nozzle clogging, low cell viability due to shear stresses, difficult to fabricate sub-micron constructs	Fibrous scaffolds only, poor mechanical properties, inefficient cellular infiltration and distribution	Only for small constructs	Limited resolution due to the deformation of PDMS stamp, substrate sagging,
Schematic	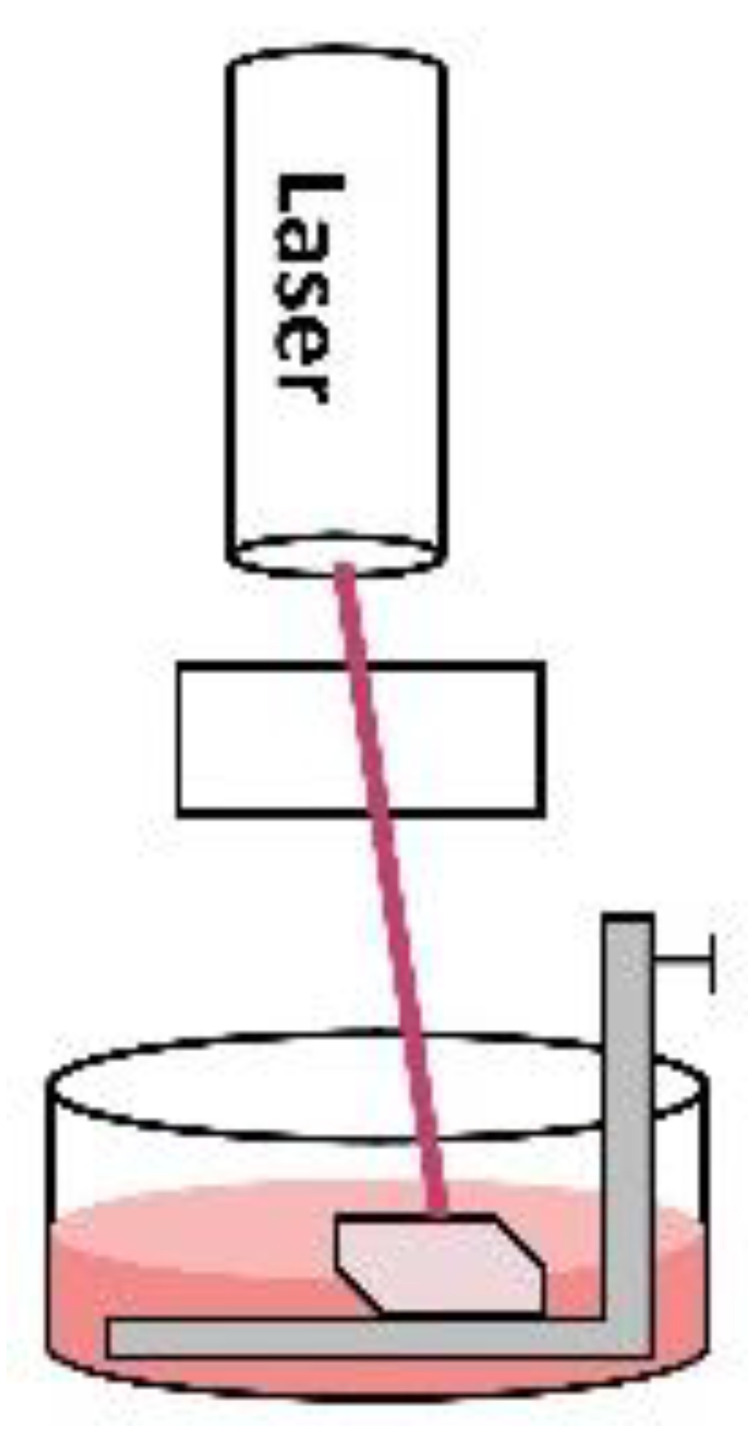	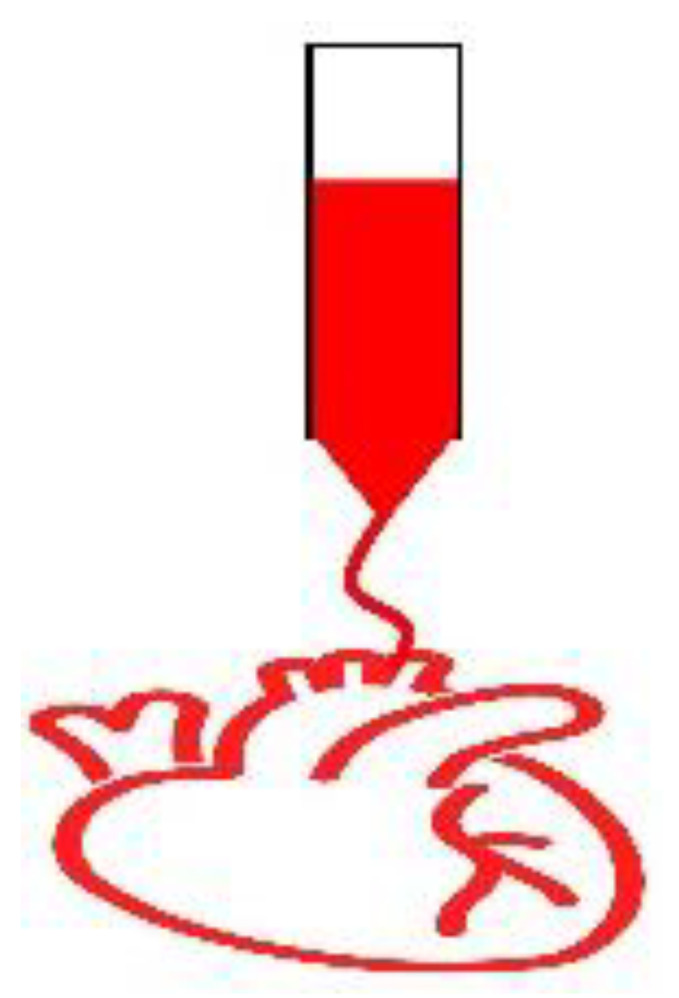	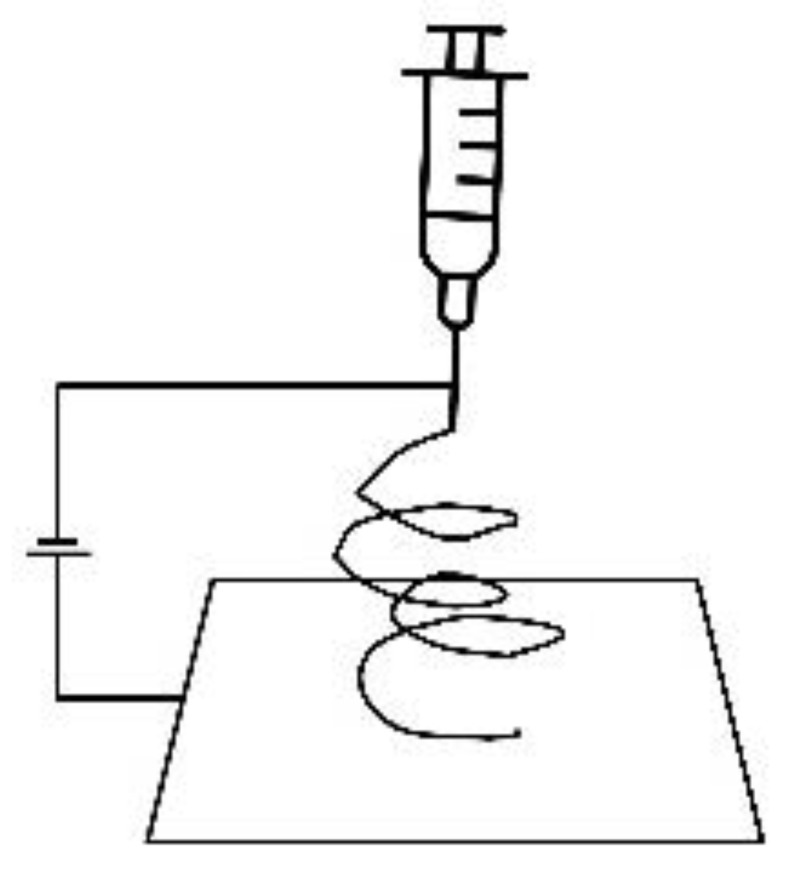	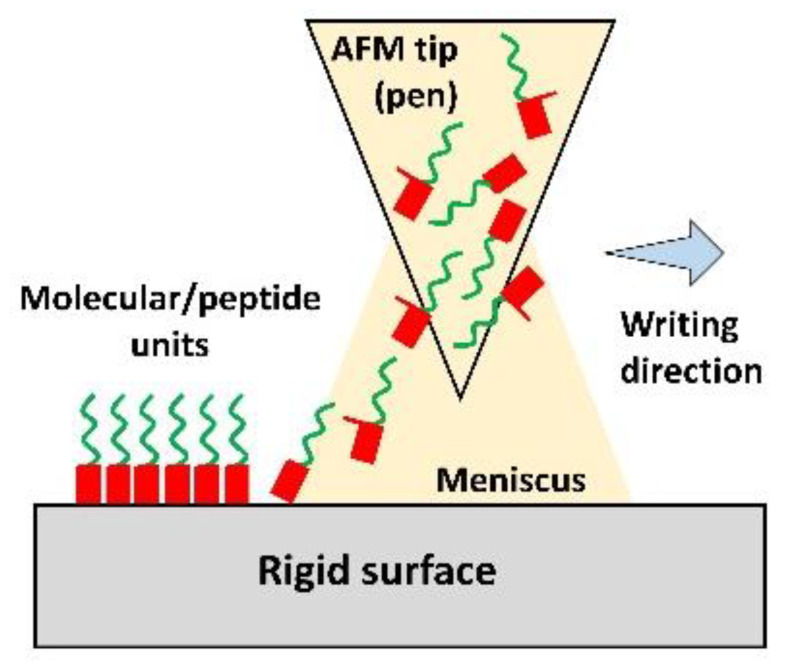	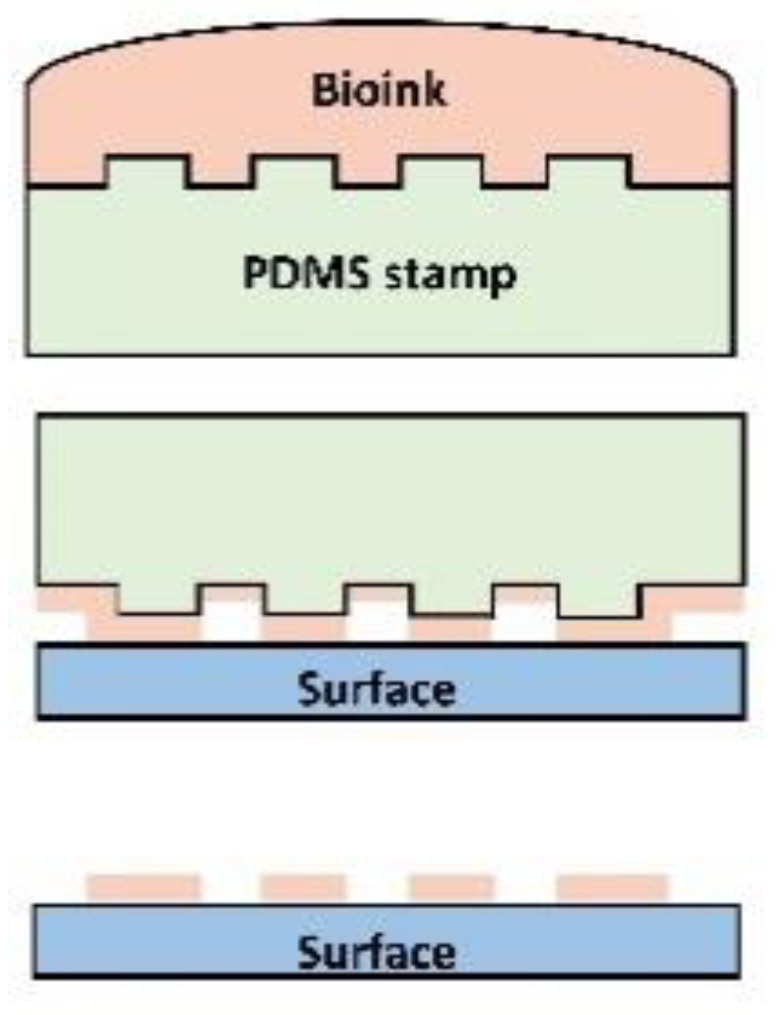
Ref	[44,47,48]	[49,59]	[60,61]	[62]	[62,63,64]

**Table 2:** Effect of conductive nano-construct on cell viability and proliferation.

Category	Conductive Construct	Mechanical Properties	Electrical Properties	Cell Line	Cellular Response
Carbon NMs	Decellularised pericardium ECM, MWCNTs hydrogels [89]	G’ = 229.25 Pa, G” = 150 Pa	Four-probe technique, σ = 15 × 10^−3^ S/cm	HL-1	Three-folds increase in the proliferation rate, enhanced expression of Cx43 and α-actinin
	PU/chitosan/ CNT membranes [83]	UTS = 21.9 MPa, E = 4.34 Mpa	Four-probe technique, R = 0.17 kΩ/sq	HUVECs, H9c2	High cell viability
	rGO foams [90]	G’ = 8 kPa	Two-probe Keithley meter, σ = 112 S/m	Neonatal rat CMs	3D organisation of CMs within the porous foam
	Collagen/CNFs nanocomposites [92]	Mechanical strength = 3.5 N	-	H9c2	Good cell viability, enhanced expression of α-actinin.
Metallic NMs	Thiol-HEMA/ GNPs hybrid hydrogels [96]	E = 0.6 MPa	Keithley electrometer, σ = 15.3 S/m	CMs	Increased viability after electrical stimulation
	GelMA/GNRs hydrogels [52]	E = 3.6 kPa	Electrochemical workstation, Z = 1 kΩ at 10^5^ Hz	CMs	Enhanced cell retention, high viability, elevated expression of Cx43, α-actinin
	Gold NPs/porcine cholecyst derived ECM [97]	-	-	H9c2	High cell viability, good proliferation rate

Abbreviations: NMs: nanomaterials, MWCNTs: multi-wall carbon nanotube, ECM: extracellular matrix, G’: shear storage modulus, G”: shear loss modulus, σ: electrical conductivity, PU: polyurethane, UTS: ultimate tensile strength, E: elastic modulus, R: electrical resistance, HUVECs: human umbilical vein endothelial cells, rGO: reduced graphene oxide, CMs: cardiomyocytes, CNF: carbon nanofibers, HEMA: hydroxyethyl methacrylate, GNPs: gold nanoparticles, GNR: gold nanorods, GelMA: gelatin methacrylate, Z: impedance.

**Table 3:** Effect of conductive nano-construct on cellular differentiation.

Category	Conductive Construct	Mechanical Properties	Electrical Properties	Cell Line	Cellular Response
Carbon NMs	MWCNTs, GelMA hydrogels [99]	E = 23.4 kPa	Z = 400 kΩ	C2C12	Myotube formation, more than two-folds increase in the expression of MRF4, α-actinin, and MHCIId/x when stimulated electrically
	CHI, PVA, MWCNTs membranes + differentiating molecules [106]	E = 941 MPa, ε_r_ = 3.8 %	Keithley Multimeter, σ= 1.2 mS/cm	USSCs	Differentiation to CMs-like cells, 3 and 58-folds increase in β-MHC and cTnI expression, respectively
	PNIAA/SWCNTs hydrogels [111]	-	R = 10 kΩ	BADSCs	Differentiation to cardiac like cells
	GelMA-CNTs hydrogels [102]	E = 30.6 kPa	CompactStat Potentiostat, Z = 56 kΩ at 0.2 Hz	129/SVE-derived mouse stem cells derived rat EBs	Three-folds increase in the expression of cTnT2 and Nkx2.5 when stimulated electrically, relatively high scaffold area covered by the beating EBs
	CNTs embedded in EBs [103]	E = 35.2 kPa	CompactStat Potentiostat, Z = 300 kΩ at 1 Hz	129/SVE-derived mouse stem cells derived rat EBs	Strong cardiac phenotype. Enhanced expression of Nkx2.5, acta2, cTnT2, MHC, MLC, Cdh5 genes
	rGO, Na-alginate hydrogels [110]	G’ = 1 kPa at ω = 10 rad/s	Four-probe technique, σ = 1/9 ± 0.16 × 10^5^ S/m	hBM-MSCs,	Readily differentiation to CMs-like cells with good viability
	Cell culture + C60-fullerene NPs [104]	-	-	BADSCs	Enhanced MAPK/ERK pathways led to differentiation to CMs-like cells, high expression of Cx43, α-actinin and cTnT
	Fullerenol/alginate hydrogels [105]	G’ = 700 Pa, G” = 100 Pa, time sweep = 0–20 sec	-	BADSCs	Enhanced MAPK/ERK pathways led to the differentiation to CMs-like cells
	Fullerene whiskers [98]	-	-	C2C12	Myotube formation, 1.4-folds increase in MyoD and myogenin expression
Metallic NMs	Gold-coated collagen nanofibers [109]	-	The Keithly instrument, ρ = 4 × 10^−5^ Ω m	Ch-MSCs	Differentiation to CMs with high proliferation rate, enhanced expression of ANP and Nkx2.5
	Gold NPs/chitosan hydrogels [108]	E_c_ = 7 kPa	Four-probe technique, σ = 0.13 S/m	MSCs	Formation of cardiomyocyte-like cells, Nkx2.5 and α-MHC upregulated by 1.80 and 2.4-folds, respectively
	PU-rGO/ Ag-NPs membranes [107]	UTS= 110 MPa, ε_r_ = 51%,	Metrohm conductometer, σ = 100 µS/cm	hCPCs	Elevated expression of Tbx18, cTnT, and α-MHC
	Gold-coated PCL membranes [100]	E = 1.69 MPa	Multi-meter, ρ = 9.5 kΩ/cm	H9c2	Myotube formation with high maturation and fusion indices, enhanced MHC expression

Abbreviations: CHI: chitosan, PVA: polyvinyl alcohol, USSCs: unrestricted somatic stem cells, BADSCs: brown adipose-derived stem cells, EBs: embryoid bodies, ω: angular frequency, hBM-MSCs: human bone marrow-derived mesenchymal stem cells, Ch-MSCs: chorion placental derived mesenchymal stem cells, hCPCs: human cardiac progenitor cells, PCL: polycaprolactone, G’: shear storage modulus, G”: shear loss modulus, ε_r_: strain at rupture, E_c_: compressive modulus, UTS: ultimate tensile strength, E = elastic modulus, ρ: electrical resistivity.

**Table 4:** Effect of conductive nano-construct on cell morphology.

Category	Conductive Construct	Mechanical Properties	Electrical Properties	Cell Line	Cellular Response
Carbon NMs	Collagen/SWCNTs composite [114]	-	Two-probe technique, σ = 1.72 × 10^−9^/Ω	NRVMs	Enhanced assembly of intercalated discs.
	rGO, Na-alginate hydrogels [110]	G’ = 1 kPa at ω = 10 rad/s	Four-probe technique, σ = 1/9 ± 0.16 × 10^5^ S/m	Neonatal rat CMs	Striated morphology with elevated expression of actn4, cTnT2, Cx43
	rGO/collagen cardiac patch [117]	E = 340 kPa	Four-probe technique, σ = 22 µS/m	CMs	Two-folds increase in actinin and Cx43 expression with five-folds increase in cTnT2
	OPF/GO hydrogels [120]	-	σ= 4.24 mS/cm	Neonatal rat cardiac fibroblasts	Well organised striated sarcomeres, enhanced expression α-tubulin, actinin, ID-related proteins
	PCL/graphene composites [119]	-	EIS, Z = 1.2 kΩ	mESCs-CMs	Contractile morphology, elevated levels of MHC, Cx43, β-actin, cTnT after 14 days
	CNF/gelatin patch [118]	UTS = 5.32 MPa, E = 8.42 MPa	Four-probe technique, σ = 84 µS/m	CMs	Cx43 and actn4 up-regulated by 3 and 4.4 folds, respectively
Metallic NMs	GelMA/GNRs Hydrogels [116]	E = 1.1 kPa	LCR meter, Z < 1 kΩ (10^2^ to 10^6^ Hz)	CMs	Increased cytoskeletal organisation, enhanced expression of Cx43, α-actinin, cTnI, synchronous beating patterns.
	Laponite loaded myocardial ECM/ gold NPs hydrogels [113]	-	-	Neonatal rat CMs	Less apoptosis rate, strong cardiac phenotype.
	Gold NPs/PCL-gelatin membranes [115]	-	-	NRVMs	Elongated and aligned morphology. High contraction amplitude
	Chitosan/Se NPs films [95]	UTS = 19 kPa, ε_r_ = 67%,	σ = 5.5 mS/cm	H9c2	Filopodia-like morphology
	Chitosan/TiO_2_ NPs hydrogels [94]	E = 1.5 MPa	-	CMs	Better cell-matrix interaction, Interconnected cardiac layers

Abbreviations: SWCNTs: single-wall carbon nanotubes, NRVMs: neonatal rat ventricular myocytes, OPF: oligo(poly(ethylene glycol) fumarate), GO: graphene oxide, ID: intercalated discs, EIS: electrochemical impedance spectroscopy, mESCs-CMs: mouse embryonic stem cells derived cardiomyocytes, Se: selenium, G’: shear storage modulus, ε_r_: strain at rupture, UTS: ultimate tensile strength, E = elastic modulus.

**Table 5:** Effect of conductive nano-construct on intercellular coupling and contractions.

Category	Conductive Construct	Mechanical Properties	Electrical Properties	Cell Line	Cellular Response
Carbon NMs	PGS-gelatin/CNTs membranes [123]	E = 373 kPa	Z = 7 kΩ at 40 Hz	Neonatal rat CMs	Enhanced Cx43 and cTnI expression, 2.2-folds increase in the beating rate after 5 days of incubation
	Pristine MWCNT films [121]	-	-	NRVMs, cardiac fibroblasts	Sarcomeric striations, tight desmosomes like nano-connections
	Pristine MWCNTs films [112]	-	σ = 3.1 mS (along fibre axes) 0.25 mS (transversely)	neonatal rat CMs	Sarcomeric striation formation, enhanced Cx43 expression, synchronised beating patterns via pacemaker
	Gelatin, chitosan, SWCNTs [122]	E_c_ = 15 kPa	-	NRVMs	Three-folds increase in the beating frequency
	PEG/PLA/CNTs membranes [124]	E = 60 Mpa, ε_r_ = 52%,	Four-probe technique, σ = 30 mS/cm	CMs	Enhanced expression of α-actinin, and cTnI. Synchronous beating at low CNT concentrations.
	PCL/CHI/Ppy/graphene patches [128]	E = 0.098 MPa, UTS = 1.27 MPa, ε_r_ = 8%	Two-probe technique, σ = 5.33 S/cm	mESCs-CMs	Enhanced cTnI expression, Beating CMs
	PEG/Graphene hybrid scaffolds [129]	-	I-V curves, R = 0.947 kΩ	NRVMs	Enhanced Cx43 expression, 2.2-folds increase in calcium transient amplitude
	GelMA/rGO hydrogels [130]	E = 22.6 kPa	Z = 1.5 kΩ at 100 Hz	Primary CMs,	Well organised striated sarcomeres, 9 times faster beating rate
	CHI, CNFs composites [125]	E = 28.1 kPa	Four-probe technique, σ = 0.25 S/m	Neonatal ratCMs, rat MI model	Strong contractile phenotype with several folds increase in Cx43, GATA4, cTnI, cTnT2, Myh6, Myh7, ANF expressions
Metallic NMs	Alginate/GNWs patch [93]	E_c_ = 3.5 kPa	C-AFM, Z < 3 kΩ at 100 kHz	Cardiac cells	Two-folds increase in Cx43, and sarcomeric α-actinin expressions
	RTG/gold NPs gels [132]	G’ = 255.3 Pa at 37 C	Multi-meter, R = 140 kΩ	NRVMs	Enhanced expression of Cx43, reduced α-actinin expression.
	Collagen/Ag-NPs membranes [133]	-	4-electrode system, σ = 0.8 µS/m	CMs	Up-regulation of Cx43 and α-actinin

Abbreviations: PGS: polyglycerol sebacate storage modulus, ANF: Atrial natriuretic factor, C-AFM: conductive atomic force microscopy, GNWs: gold nanowires, RTG: reverse thermal gels, G’: shear storage modulus, ε_r_: strain at rupture, E_c_: compressive modulus, UTS: ultimate tensile strength, E = elastic modulus.

**Table 6:** Conductive nanomaterials as delivery vehicles for cardiac gene therapy.

Nanomaterials	Delivered Gene	Outcomes
AuNPs [134]	Deoxyribozyme (DNAzyme)	Knockdown of 50% TNF-α expression. Improved anti-inflammatory pathways
AuNPs [135]	Antago-miR155	Improved blood pumping ability
AuNPs [136]	Circ-Amolt1	Cardio-protection against Doxorubicin-induced cardiomyopathy
SWCNTs [137]	siRNA/Caspase3	Casepase3 silencing, 1.42-folds increase in the infarcted wall thickness with reduced scar size
Graphene [138]	DNA_VEGF_	Improved angiogenesis, better cardiac performance

Abbreviations: TNF: tumour necrosis factor, VEGF: vascular endothelial growth factor, DNA: deoxyribonucleic acid, AuNPs: gold nanoparticles, SWCNTs: single-wall carbon nanotubes.

**Table 7:** Preclinical studies using rat MI models to repair the ischemic tissue.

Conductive Construct	Outcomes
Gelatin/SWCNTs hydrogels [141]	**Hydrogel injected heart:** EF/FS improved to 49%/21.9%, enhanced expression of ILK, p-AKT, β1-integrin, and β-catenin after four weeks. **Gelatin injected heart:** Reduced EF/FS of 43.4%/18.8%, expression of the above genes was not very pronounced.
PNIAA/SWCNTs hydrogels + BADSCs [111]	**Hydrogel + BADSCs injected heart:** Improved blood pumping ability and LV wall regenerated to 863 µm, infarct size reduced by two-folds, more cells could survive the hostile MI environment after four weeks. **PBS injected heart:** Poor blood pumping ability with larger scar size with large infarct size, thin LV wall of 538 µm.
PEG-MEL/HA-SH/GO composites [140]	**Scaffold implanted heart:** Improved tissue regeneration with LV wall thickness of 1.9 mm, scar size reduced to 37% from 52.5%, four weeks post-injection. **PBS injected heart:** LV wall was around 0.9 mm thick, blood-pumping ability dropped significantly.
SF, GO hydrogels [139]	**Hydrogel injected heart:** LV wall thickness increased to 280 µm, reduced infarct size with 1.8-folds decrease in relative scar thickness. **MI heart:** LV wall thickness was around 250 µm, larger infarct size.
OPF/GO hydrogels [120]	**Hydrogel injected heart:** LV wall regenerated to 0.77 mm, infarct size reduced by 1.6-folds, improved blood pumping ability with reduced left ventricular diameter at end-systole and at end-diastole, high infiltration of macrophages, two weeks post-injection. **PBS injected heart:** Thin LV wall around 0.37 mm, larger infarct size with reduced ejection fraction and fraction shortening.
Fullerenol/alginate hydrogels [105]	**Hydrogel + BADSCs injected heart:** Improved angiogenesis with twice the vessel density, 1.3 mm thick LV wall, decreased left ventricular internal diameter at end-systole and at end-diastole, four weeks post-injection. **PBS + BADSCs injected heart:** Least angiogenesis with reduced blood pumping ability, 0.61 mm thick LV wall, wider left ventricular internal diameter at end-systole and at end-diastole.
Collagen/CNFs composites [92]	**Scaffold implanted heart:** Improved regeneration of the LV wall with sarcomeric morphology and high angiogenesis. **MI heart:** Damaged intercalated discs assembly, high tissue degeneration.

Abbreviations: EF/FS: Ejection fraction/Fraction shortening, LV: Left ventricle, OPF: oligo(poly(ethylene glycol) fumarate), PEG-MEL/HA-SH: Polyethylene glycol-melamine/Thiol modified hyaluronic acid, SF: Silk fibroin, PNIAA: poly(N-isopropylacrylamide).

**Table 8:** The best possible strategy to resolve different cardiac-related issues using conductive nanomaterials.

Conductive Nanomaterial	Approach	Major Issue Resolved	Studies
SWCNTs, GO, Fullerene, AuNPs	In-vivo scaffold implantation, gene delivery	Blood pumping ability	[105,120,135,141]
Fullerene, CNFs, Graphene	In-vivo scaffold implantation, gene delivery	In-vivo angiogenesis	[92,105,138]
SWCNTs, GO	In-vivo scaffold implantation, gene delivery	LV wall regeneration and reduced scar size	[111,120,139,140]
AuNPs	Gene delivery	Chemotherapy-induced cardiomyopathy	[136]
SWCNTs, AuNPs	In-vivo scaffold implantation, gene delivery	Inflammation	[111,134,137]

## References

[1] Roth G.A., Mensah G.A., Johnson C.O., Addolorato G., Ammirati E., Baddour L.M., Barengo N.C., Beaton A.Z., Benjamin E.J., Benziger C.P. (2020). Global Burden of Cardiovascular Diseases and Risk Factors, 1990–2019: Update From the GBD 2019 Study. J. Am. Coll. Cardiol..

[2] Timmis A., Townsend N., Gale C.P., Torbica A., Lettino M., Petersen S.E., Mossialos E.A., Maggioni A.P., Kazakiewicz D., May H.T. (2020). European Society of Cardiology: Cardiovascular Disease Statistics 2019. Eur. Heart J..

[3] Wilkins E., Wilson L., Wickramasinghe K., Bhatnagar P. (2017). European Cardiovascular Disease Statistics 2017. Eur. Heart Netw..

[4] Ambrose J.A., Singh M. (2015). Pathophysiology of coronary artery disease leading to acute coronary syndromes. F1000Prime Rep..

[5] Bergmann O., Zdunek S., Felker A., Salehpour M., Alkass K., Bernard S., Sjostrom S.L., Szewczykowska M., Jackowska T., Dos Remedios C. (2015). Dynamics of Cell Generation and Turnover in the Human Heart. Cell.

[6] Sadahiro T. (2019). Cardiac regeneration with pluripotent stem cell-derived cardiomyocytes and direct cardiac reprogramming. Regen. Ther..

[7] Frangogiannis N.G. (2015). Pathophysiology of Myocardial Infarction. Compr. Physiol..

[8] Birks E.J., Tansley P.D., Hardy J., George R., Bowles C.T., Burke M., Banner N.R., Khaghani A., Yacoub M.H. (2006). Left Ventricular Assist Device and Drug Therapy for the Reversal of Heart Failure. N. Engl. J. Med..

[9] Bangalore S., Fakheri R., Wandel S., Toklu B., Wandel J., Messerli F.H. (2017). Renin angiotensin system inhibitors for patients with stable coronary artery disease without heart failure: Systematic review and meta-analysis of randomized trials. BMJ.

[10] Freemantle N., Cleland J., Young P., Mason J., Harrison J. (1999). β Blockade after myocardial infarction: Systematic review and meta regression analysis. Br. Med. J..

[11] Zhang Q., Xiang J., Wang X., Liu H., Hu B., Feng M., Fu Q. (2010). β2-adrenoceptor agonist clenbuterol reduces infarct size and myocardial apoptosis after myocardial ischaemia/reperfusion in anaesthetized rats. Br. J. Pharmacol..

[12] Tian Y., Miao B., Charles E.J., Wu D., Kron I.L., French B.A., Yang Z. (2018). Stimulation of the Beta2 Adrenergic Receptor at Reperfusion Limits Myocardial Reperfusion Injury via an Interleukin-10-Dependent Anti-Inflammatory Pathway in the Spleen. Circ. J..

[13] Slaughter M.S., Rogers J.G., Milano C.A., Russell S.D., Conte J.V., Feldman D., Sun B., Tatooles A.J., Delgado R.M., Long J.W. (2009). Advanced Heart Failure Treated with Continuous-Flow Left Ventricular Assist Device. N. Engl. J. Med..

[14] Cheng J.M., Uil C.A.D., Hoeks S.E., Van Der Ent M., Jewbali L.S., Van Domburg R.T., Serruys P.W. (2009). Percutaneous left ventricular assist devices vs. intra-aortic balloon pump counterpulsation for treatment of cardiogenic shock: A meta-analysis of controlled trials. Eur. Heart J..

[15] Starling R.C., Moazami N., Silvestry S.C., Ewald G., Rogers J.G., Milano C.A., Rame J.E., Acker M.A., Blackstone E.H., Ehrlinger J. (2014). Unexpected Abrupt Increase in Left Ventricular Assist Device Thrombosis. N. Engl. J. Med..

[16] Zammaretti P., Jaconi M. (2004). Cardiac tissue engineering: Regeneration of the wounded heart. Curr. Opin. Biotechnol..

[17] Wang Z., Lee S.J., Cheng H.-J., Yoo J.J., Atala A. (2018). 3D bioprinted functional and contractile cardiac tissue constructs. Acta Biomater..

[18] Eschenhagen T., Didié M., Münzel F., Schubert P., Schneiderbanger K., Zimmermann W.H. (2002). 3D engineered heart tissue for replacement therapy. Basic Res. Cardiol. Suppl..

[19] Zimmermann W.-H., Melnychenko I., Wasmeier G.H., Didié M., Naito H., Nixdorff U., Hess A., Budinsky L., Brune K., Michaelis B. (2006). Engineered heart tissue grafts improve systolic and diastolic function in infarcted rat hearts. Nat. Med..

[20] Schwan J., Kwaczala A.T., Ryan T.J., Bartulos O., Ren Y., Sewanan L., Morris A.H., Jacoby D.L., Qyang Y., Campbell S.G. (2016). Anisotropic engineered heart tissue made from laser-cut decellularized myocardium. Sci. Rep..

[21] Zimmermann W.-H., Melnychenko I., Eschenhagen T. (2004). Engineered heart tissue for regeneration of diseased hearts. Biomaterials.

[22] Ciocci M., Mochi F., Carotenuto F., Di Giovanni E., Prosposito P., Francini R., De Matteis F., Reshetov I.V., Casalboni M., Melino S. (2017). Scaffold-in-Scaffold Potential to Induce Growth and Differentiation of Cardiac Progenitor Cells. Stem Cells Dev..

[23] Asgari M., Latifi N., Heris H.K., Vali H., Mongeau L. (2017). In vitro fibrillogenesis of tropocollagen type III in collagen type I affects its relative fibrillar topology and mechanics. Sci. Rep..

[24] Pappano A.J., Gil Wier W. (2013). Automaticity: Natural excitation of the heart. Cardiovascular Physiology.

[25] Ali Q., Malik S., Malik A., Hafeez M.N., Salman S. (2020). Role of Modern Technologies in Tissue Engineering. Arch. Neurosci..

[26] Quijada C. (2020). Special Issue: Conductive Polymers: Materials and Applications. Materials.

[27] Yi N., Abidian M.R. (2016). Conducting polymers and their biomedical applications. Biosynthetic Polymers for Medical Applications.

[28] Lindsey M.L., Jung M., Hall M.E., DeLeon-Pennell K.Y. (2018). Proteomic analysis of the cardiac extracellular matrix: Clinical research applications. Expert Rev. Proteom..

[29] Silva A.C., Pereira C., Fonseca A.C.R.G., Pinto-Do-Ó P., Nascimento D.S. (2021). Bearing My Heart: The Role of Extracellular Matrix on Cardiac Development, Homeostasis, and Injury Response. Front. Cell Dev. Biol..

[30] Chang S.-W., Buehler M.J. (2014). Molecular biomechanics of collagen molecules. Mater. Today.

[31] Ushiki T. (2002). Collagen Fibers, Reticular Fibers and Elastic Fibers. A Comprehensive Understanding from a Morphological Viewpoint. Arch. Histol. Cytol..

[32] Engel J., Odermatt E., Engel A., Madri J.A., Furthmayr H., Rohde H., Timpl R. (1981). Shapes, domain organizations and flexibility of laminin and fibronectin, two multifunctional proteins of the extracellular matrix. J. Mol. Biol..

[33] Antman E., Bassand J.-P., Klein W., Ohman M., Sendon J.L.L., Rydén L., Simoons M., Tendera M. (2000). Myocardial Infarction Redefined--a Consensus Document of The Joint European Society of Cardiology/American College of Cardiology Committee for the Redefinition of Myocardial Infarction. J. Am. Coll. Cardiol..

[34] Nalbantgil I. (2002). Ventricular arrhythmias in hypertensive patients. Anadolu Kardiyol. Derg.

[35] Mewton N., Liu C.Y., Croisille P., Bluemke D., Lima J.A. (2011). Assessment of Myocardial Fibrosis With Cardiovascular Magnetic Resonance. J. Am. Coll. Cardiol..

[36] Janicki J.S., Brower G.L. (2002). The role of myocardial fibrillar collagen in ventricular remodeling and function. J. Card. Fail..

[37] DeLeon-Pennell K.Y., Meschiari C.A., Jung M., Lindsey M.L. (2017). Matrix Metalloproteinases in Myocardial Infarction and Heart Failure.

[38] Stuart S.F., De Jesus N.M., Lindsey M., Ripplinger C.M. (2016). The crossroads of inflammation, fibrosis, and arrhythmia following myocardial infarction. J. Mol. Cell. Cardiol..

[39] Ma Y., Brás L.E.D.C., Toba H., Iyer R.P., Hall M.E., Winniford M.D., Lange R.A., Tyagi S.C., Lindsey M.L. (2014). Myofibroblasts and the extracellular matrix network in post-myocardial infarction cardiac remodeling. Pflug. Arch. Eur. J. Physiol..

[40] Yabluchanskiy A., Ma Y., Iyer R.P., Hall M.E., Lindsey M.L. (2013). Matrix Metalloproteinase-9: Many Shades of Function in Cardiovascular Disease. Physiology.

[41] Lindsey M.L., Escobar G.P., Dobrucki L.W., Goshorn D.K., Bouges S., Mingoia J.T., McClister D.M., Su H., Gannon J., MacGillivray C. (2006). Matrix metalloproteinase-9 gene deletion facilitates angiogenesis after myocardial infarction. Am. J. Physiol. Heart Circ. Physiol..

[42] Frodermann V., Nahrendorf M. (2016). Neutrophil–macrophage cross-talk in acute myocardial infarction. Eur. Heart J..

[43] Ma Y., Halade G.V., Lindsey M.L. (2012). Extracellular matrix and fibroblast communication following myocardial infarction. J. Cardiovasc. Transl. Res..

[44] Emons M., Obata K., Binhammer T., Ovsianikov A., Chichkov B.N., Morgner U. (2012). Two-photon polymerization technique with sub-50 nm resolution by sub-10 fs laser pulses. Opt. Mater. Express.

[45] Gao L., Kupfer M.E., Jung J.P., Yang L., Zhang P., Da Sie Y., Tran Q., Ajeti V., Freeman B.T., Fast V.G. (2017). Myocardial Tissue Engineering With Cells Derived From Human-Induced Pluripotent Stem Cells and a Native-Like, High-Resolution, 3-Dimensionally Printed Scaffold. Circ. Res..

[46] Fang F., Aabith S., Homer-Vanniasinkam S., Tiwari M.K. (2017). High-Resolution 3D Printing for Healthcare Underpinned by Small-Scale Fluidics.

[47] Zheng X., Lee H., Weisgraber T.H., Shusteff M., DeOtte J., Duoss E.B., Kuntz J.D., Biener M.M., Ge Q., Jackson J.A. (2014). Ultralight, ultrastiff mechanical metamaterials. Science.

[48] Ge Q., Li Z., Wang Z., Kowsari K., Zhang W., He X., Zhou J., Fang N.X. (2020). Projection micro stereolithography based 3D printing and its applications. Int. J. Extreme Manuf..

[49] Miri A.K., Mirzaee I., Hassan S., Oskui S.M., Nieto D., Khademhosseini A., Zhang Y.S. (2019). Effective bioprinting resolution in tissue model fabrication. Lab Chip.

[50] Murphy S.V., Atala A. (2014). 3D bioprinting of tissues and organs. Nat. Biotechnol..

[51] Izadifar M., Chapman D., Babyn P., Chen X., Kelly M. (2018). UV-Assisted 3D Bioprinting of Nanoreinforced Hybrid Cardiac Patch for Myocardial Tissue Engineering. Tissue Eng. Part C Methods.

[52] Zhu K., Shin S.R., van Kempen T., Li Y.-C., Ponraj V., Nasajpour A., Mandla S., Hu N., Liu X., Leijten J. (2017). Gold Nanocomposite Bioink for Printing 3D Cardiac Constructs. Adv. Funct. Mater..

[53] Ho C.M.B., Mishra A., Lin P.T.P., Ng S.H., Yeong W.Y., Kim Y.-J., Yoon Y.-J. (2017). 3D Printed Polycaprolactone Carbon Nanotube Composite Scaffolds for Cardiac Tissue Engineering. Macromol. Biosci..

[54] Jung D., Minami I., Patel S., Lee J., Jiang B., Yuan Q., Li L., Kobayashi S., Chen Y., Lee K.-B. (2012). Incorporation of Functionalized Gold Nanoparticles into Nanofibers for Enhanced Attachment and Differentiation of Mammalian Cells. J. Nanobiotechnol..

[55] Crowder S.W., Liang Y., Rath R., Park A.M., Maltais S., Pintauro P.N., Hofmeister W., Lim C.C., Wang X., Sung H.-J. (2013). Poly(ε-caprolactone)–carbon nanotube composite scaffolds for enhanced cardiac differentiation of human mesenchymal stem cells. Nanomedicine.

[56] Ravichandran R., Sridhar R., Venugopal J.R., Sundarrajan S., Mukherjee S., Ramakrishna S. (2014). Gold Nanoparticle Loaded Hybrid Nanofibers for Cardiogenic Differentiation of Stem Cells for Infarcted Myocardium Regeneration. Macromol. Biosci..

[57] Birchall L., Qu H., Ulijn R. (2011). Surface modification of biomaterials by peptide functionalisation. Surf. Modif. Biomater. Methods Anal. Appl..

[58] Atmanli A., Domian I.J. (2013). Generation of Aligned Functional Myocardial Tissue Through Microcontact Printing. J. Vis. Exp..

[59] Kang H.-W., Lee S.J., Ko I.K., Kengla C., Yoo J.J., Atala A. (2016). A 3D bioprinting system to produce human-scale tissue constructs with structural integrity. Nat. Biotechnol..

[60] Tourlomousis F., Ding H., Dole A., Chang R.C. Towards resolution enhancement and process repeatability with a melt electrospinning writing process: Design and protocol considerations. Proceedings of the ASME 2016 11th International Manufacturing Science and Engineering Conference.

[61] Hong J., Yeo M., Yang G.H., Kim G. (2019). Cell-Electrospinning and Its Application for Tissue Engineering. Int. J. Mol. Sci..

[62] Cassidy J. (2014). Nanotechnology in the Regeneration of Complex Tissues. Bone Tissue Regen. Insights.

[63] Hannachi I., Itoga K., Kumashiro Y., Kobayashi J., Yamato M., Okano T. (2009). Fabrication of transferable micropatterned-co-cultured cell sheets with microcontact printing. Biomaterials.

[64] Perl A., Reinhoudt D.N., Huskens J. (2009). Microcontact Printing: Limitations and Achievements. Adv. Mater..

[65] Santana L.F., Cheng E.P., Lederer W.J. (2010). How does the shape of the cardiac action potential control calcium signaling and contraction in the heart?. J. Mol. Cell. Cardiol..

[66] Stoppel W.L., Kaplan D.L., Black L.D. (2016). Electrical and mechanical stimulation of cardiac cells and tissue constructs. Adv. Drug Deliv. Rev..

[67] Passier R., Zeng H., Frey N., Naya F.J., Nicol R.L., McKinsey T.A., Overbeek P., Richardson J.A., Grant S.R., Olson E.N. (2000). CaM kinase signaling induces cardiac hypertrophy and activates the MEF2 transcription factor in vivo. J. Clin. Investig..

[68] Adachi A., Takahashi T., Ogata T., Imoto-Tsubakimoto H., Nakanishi N., Ueyama T., Matsubara H. (2012). NFAT5 regulates the canonical Wnt pathway and is required for cardiomyogenic differentiation. Biochem. Biophys. Res. Commun..

[69] Chen Y., Cao X. (2009). NFAT directly regulates Nkx2-5 transcription during cardiac cell differentiation. Biol. Cell.

[70] Xia Y., McMillin J.B., Lewis A., Moore M., Zhu W.G., Williams R.S., Kellems R.E. (2000). Electrical Stimulation of Neonatal Cardiac Myocytes Activates the NFAT3 and GATA4 Pathways and Up-regulates the Adenylosuccinate Synthetase 1 Gene. J. Biol. Chem..

[71] Kotini M., Barriga E.H., Leslie J., Gentzel M., Rauschenberger V., Schambony A., Mayor R. (2018). Gap junction protein Connexin-43 is a direct transcriptional regulator of N-cadherin in vivo. Nat. Commun..

[72] Watt A.J., Battle M.A., Li J., Duncan S.A. (2004). GATA4 is essential for formation of the proepicardium and regulates cardiogenesis. Proc. Natl. Acad. Sci. USA.

[73] Saadane N., Alpert L., Chalifour L.E. (1999). Expression of immediate early genes, GATA-4, and Nkx-2.5 in adrenergic-induced cardiac hypertrophy and during regression in adult mice. Br. J. Pharmacol..

[74] Wei B., Jin J.-P. (2016). TNNT1, TNNT2, and TNNT3: Isoform genes, regulation, and structure–function relationships. Gene.

[75] Estigoy C.B., Pontén F., Odeberg J., Herbert B., Guilhaus M., Charleston M., Ho J.W.K., Cameron D., Dos Remedios C.G. (2009). Intercalated discs: Multiple proteins perform multiple functions in non-failing and failing human hearts. Biophys. Rev..

[76] Zhao G., Qiu Y., Zhang H.M., Yang D. (2019). Intercalated discs: Cellular adhesion and signaling in heart health and diseases. Heart Fail. Rev..

[77] Liu X., George M., Parkc S., Ii A.L.M., Gaihreab B., Liab L., Waletzki B.E., Terzicc A., Yaszemskiab M.J., Luab L. (2020). 3D-printed scaffolds with carbon nanotubes for bone tissue engineering: Fast and homogeneous one-step functionalization. Acta Biomater..

[78] Zadeh Z.E., Solouk A., Shafieian M., Nazarpak M.H. (2021). Electrospun polyurethane/carbon nanotube composites with different amounts of carbon nanotubes and almost the same fiber diameter for biomedical applications. Mater. Sci. Eng. C.

[79] Liu X., Gaihre B., George M., Miller A.L., Xu H., Waletzki B.E., Lu L. (2021). 3D bioprinting of oligo(poly[ethylene glycol] fumarate) for bone and nerve tissue engineering. J. Biomed. Mater. Res. Part A.

[80] Fischer K., Flagg D.H., Freeman J.W. (2011). Coaxial electrospun poly(ε-caprolactone), multiwalled carbon nanotubes, and polyacrylic acid/polyvinyl alcohol scaffold for skeletal muscle tissue engineering. J. Biomed. Mater. Res. Part A.

[81] Martinelli V., Bosi S., Peña B., Baj G., Long C.S., Sbaizero O., Giacca M., Prato M., Mestroni L., Peña B. (2018). 3D Carbon-Nanotube-Based Composites for Cardiac Tissue Engineering. ACS Appl. Bio Mater..

[82] Peña B., Bosi S., Aguado B.A., Borin D., Farnsworth N., Dobrinskikh E., Rowland T.J., Martinelli V., Jeong M., Taylor M.R.G. (2017). Injectable Carbon Nanotube-Functionalized Reverse Thermal Gel Promotes Cardiomyocytes Survival and Maturation. ACS Appl. Mater. Interfaces.

[83] Ahmadi P., Nazeri N., Derakhshan M.A., Ghanbari H. (2021). Preparation and characterization of polyurethane/chitosan/CNT nanofibrous scaffold for cardiac tissue engineering. Int. J. Biol. Macromol..

[84] Ma J., Wang J.-N., Tsai C.-J., Nussinov R., Ma B. (2010). Diameters of single-walled carbon nanotubes (SWCNTs) and related nanochemistry and nanobiology. Signal Image Video Process..

[85] Sakurai S., Inaguma M., Futaba D.N., Yumura M., Hata K. (2013). A Fundamental Limitation of Small Diameter Single-Walled Carbon Nanotube Synthesis—A Scaling Rule of the Carbon Nanotube Yield with Catalyst Volume. Materials.

[86] Lanone S., Andujar P., Kermanizadeh A., Boczkowski J. (2013). Determinants of carbon nanotube toxicity. Adv. Drug Deliv. Rev..

[87] Saito N., Haniu H., Usui Y., Aoki K., Hara K., Takanashi S., Shimizu M., Narita N., Okamoto M., Kobayashi S. (2014). Safe Clinical Use of Carbon Nanotubes as Innovative Biomaterials. Chem. Rev..

[88] Chen B.T., Schwegler-Berry D., McKinney W., Stone S., Cumpston J.L., Friend S., Porter D.W., Castranova V., Frazer D.G. (2012). Multi-walled carbon nanotubes: Sampling criteria and aerosol characterization. Inhal. Toxicol..

[89] Roshanbinfar K., Hilborn J., Varghese O.P., Oommen O.P. (2017). Injectable and thermoresponsive pericardial matrix derived conductive scaffold for cardiac tissue engineering. RSC Adv..

[90] Wang Y., Dong Y., Chen P., Chen R., Li Y., Du W., Liu B.-F. (2020). Reduced graphene oxide foam templated by nickel foam for organ-on-a-chip engineering of cardiac constructs. Mater. Sci. Eng. C.

[91] Lang Q., Wu Y., Ren Y., Tao Y., Lei L., Jiang H. (2015). AC Electrothermal Circulatory Pumping Chip for Cell Culture. ACS Appl. Mater. Interfaces.

[92] Tashakori-Miyanroudi M., Rakhshan K., Ramez M., Asgarian S., Janzadeh A., Azizi Y., Seifalian A., Ramezani F. (2020). Conductive carbon nanofibers incorporated into collagen bio-scaffold assists myocardial injury repair. Int. J. Biol. Macromol..

[93] Dvir T., Timko B., Brigham M., Naik S.R., Karajanagi S.S., Levy O., Jin H., Parker K.K., Langer R., Kohane D.S. (2011). Nanowired three-dimensional cardiac patches. Nat. Nanotechnol..

[94] Liu N., Chen J., Zhuang J., Zhu P. (2018). Fabrication of engineered nanoparticles on biological macromolecular (PEGylated chitosan) composite for bio-active hydrogel system in cardiac repair applications. Int. J. Biol. Macromol..

[95] Kalishwaralal K., Jeyabharathi S., Sundar K., Selvamani S., Prasanna M., Muthukumaran A. (2018). A novel biocompatible chitosan–Selenium nanoparticles (SeNPs) film with electrical conductivity for cardiac tissue engineering application. Mater. Sci. Eng. C.

[96] You J.-O., Rafat M., Ye G.J.C., Auguste D.T. (2011). Nanoengineering the Heart: Conductive Scaffolds Enhance Connexin 43 Expression. Nano Lett..

[97] Nair R.S., Ameer J.M., Alison M.R., Anilkumar T.V. (2017). A gold nanoparticle coated porcine cholecyst-derived bioscaffold for cardiac tissue engineering. Colloids Surfaces B Biointerfaces.

[98] Minami K., Kasuya Y., Yamazaki T., Ji Q., Nakanishi W., Hill J., Sakai H., Ariga K. (2015). Highly Ordered 1D Fullerene Crystals for Concurrent Control of Macroscopic Cellular Orientation and Differentiation toward Large-Scale Tissue Engineering. Adv. Mater..

[99] Ramón-Azcón J., Ahadian S., Estili M., Liang X., Ostrovidov S., Kaji H., Shiku H., Ramalingam M., Nakajima K., Sakka Y. (2013). Dielectrophoretically Aligned Carbon Nanotubes to Control Electrical and Mechanical Properties of Hydrogels to Fabricate Contractile Muscle Myofibers. Adv. Mater..

[100] Zhang Y., Zhang Z., Wang Y., Su Y., Chen M. (2020). 3D myotube guidance on hierarchically organized anisotropic and conductive fibers for skeletal muscle tissue engineering. Mater. Sci. Eng. C.

[101] Hwang Y.-S., Chung B.G., Ortmann D., Hattori N., Moeller H.-C., Khademhosseini A. (2009). Microwell-mediated control of embryoid body size regulates embryonic stem cell fate via differential expression of WNT5a and WNT11. Proc. Natl. Acad. Sci. USA.

[102] Ahadian S., Yamada S., Ramón-Azcón J., Estili M., Liang X., Nakajima K., Shiku H., Khademhosseini A., Matsue T. (2016). Hybrid hydrogel-aligned carbon nanotube scaffolds to enhance cardiac differentiation of embryoid bodies. Acta Biomater..

[103] Ahadian S., Yamada S., Estili M., Liang X., Sadeghian R.B., Nakajima K., Shiku H., Matsue T., Khademhosseini A. (2017). Carbon nanotubes embedded in embryoid bodies direct cardiac differentiation. Biomed. Microdevices.

[104] Hao T., Zhou J., Lü S., Yang B., Wang Y., Fang W., Jiang X., Lin Q., Li J., Wang C. (2016). Fullerene Mediates Proliferation and Cardiomyogenic Differentiation of Adipose-Derived Stem Cells via Modulation of MAPK Pathway and Cardiac Protein Expression. Int. J. Nanomed..

[105] Hao T., Li J., Yao F., Dong D., Wang Y., Yang B., Wang C. (2017). Injectable Fullerenol/Alginate Hydrogel for Suppression of Oxidative Stress Damage in Brown Adipose-Derived Stem Cells and Cardiac Repair. ACS Nano.

[106] Abedi A., Bakhshandeh B., Babaie A., Mohammadnejad J., Vahdat S., Mombeiny R., Moosavi S.R., Amini J., Tayebi L. (2021). Concurrent application of conductive biopolymeric chitosan/ polyvinyl alcohol/ MWCNTs nanofibers, intracellular signaling manipulating molecules and electrical stimulation for more effective cardiac tissue engineering. Mater. Chem. Phys..

[107] Nazari H., Azadi S., Hatamie S., Zomorrod M.S., Ashtari K., Soleimani M., Hosseinzadeh S. (2019). Fabrication of graphene-silver/polyurethane nanofibrous scaffolds for cardiac tissue engineering. Polym. Adv. Technol..

[108] Baei P., Firoozinezhad S.J., Rajabi-Zeleti S., Tafazzoli-Shadpour M., Baharvand H., Aghdami N. (2016). Electrically conductive gold nanoparticle-chitosan thermosensitive hydrogels for cardiac tissue engineering. Mater. Sci. Eng. C.

[109] Orza A., Soritau O., Olenic L., Diudea M., Florea A., Ciuca D.R., Mihu C., Casciano D., Biris A.S. (2011). Electrically Conductive Gold-Coated Collagen Nanofibers for Placental-Derived Mesenchymal Stem Cells Enhanced Differentiation and Proliferation. ACS Nano.

[110] Hajishoreh N.K., Baheiraei N., Naderi N., Salehnia M. (2020). Reduced graphene oxide facilitates biocompatibility of alginate for cardiac repair. J. Bioact. Compat. Polym..

[111] Li X., Zhou J., Liu Z., Chen J., Lü S., Sun H., Li J., Lin Q., Yang B., Duan C. (2014). A PNIPAAm-based thermosensitive hydrogel containing SWCNTs for stem cell transplantation in myocardial repair. Biomaterials.

[112] Ren J., Xu Q., Chen X., Li W., Guo K., Zhao Y., Wang Q., Zhang Z., Peng H., Li Y.-G. (2017). Superaligned Carbon Nanotubes Guide Oriented Cell Growth and Promote Electrophysiological Homogeneity for Synthetic Cardiac Tissues. Adv. Mater..

[113] Zhang Y., Fan W., Wang K., Wei H., Zhang R., Wu Y. (2019). Novel preparation of Au nanoparticles loaded Laponite nanoparticles/ECM injectable hydrogel on cardiac differentiation of resident cardiac stem cells to cardiomyocytes. J. Photochem. Photobiol. B Biol..

[114] Sun H., Lü S., Jiang X.-X., Li X., Li H., Lin Q., Mou Y., Zhao Y., Han Y., Zhou J. (2015). Carbon nanotubes enhance intercalated disc assembly in cardiac myocytes via the β1-integrin-mediated signaling pathway. Biomaterials.

[115] Shevach M., Maoz B.M., Feiner R., Shapira A., Dvir T. (2013). Nanoengineering gold particle composite fibers for cardiac tissue engineering. J. Mater. Chem. B.

[116] Navaei A., Saini H., Christenson W., Sullivan R.T., Ros R., Nikkhah M. (2016). Gold nanorod-incorporated gelatin-based conductive hydrogels for engineering cardiac tissue constructs. Acta Biomater..

[117] Norahan M.H., Pourmokhtari M., Saeb M.R., Bakhshi B., Zomorrod M.S., Baheiraei N. (2019). Electroactive cardiac patch containing reduced graphene oxide with potential antibacterial properties. Mater. Sci. Eng. C.

[118] Mehrabi A., Baheiraei N., Adabi M., Amirkhani Z. (2019). Development of a Novel Electroactive Cardiac Patch Based on Carbon Nanofibers and Gelatin Encouraging Vascularization. Appl. Biochem. Biotechnol..

[119] Hitscherich P., Aphale A., Gordan R., Whitaker R., Singh P., Xie L.-H., Patra P., Lee E.J. (2018). Electroactive graphene composite scaffolds for cardiac tissue engineering. J. Biomed. Mater. Res. Part A.

[120] Zhou J., Yang X., Liu W., Wang C., Shen Y., Zhang F., Zhu H., Sun H., Chen J., Lam J. (2018). Injectable OPF/graphene oxide hydrogels provide mechanical support and enhance cell electrical signaling after implantation into myocardial infarct. Theranostics.

[121] Martinelli V., Cellot G., Toma F.M., Long C., Caldwell J.H., Zentilin L., Giacca M., Turco A., Prato M., Ballerini L. (2012). Carbon Nanotubes Promote Growth and Spontaneous Electrical Activity in Cultured Cardiac Myocytes. Nano Lett..

[122] Pok S., Vitale F., Eichmann S.L., Benavides O.M., Pasquali M., Jacot J.G. (2014). Biocompatible Carbon Nanotube–Chitosan Scaffold Matching the Electrical Conductivity of the Heart. ACS Nano.

[123] Kharaziha M., Shin S.R., Nikkhah M., Topkaya S.N., Masoumi N., Annabi N., Dokmeci M.R., Khademhosseini A. (2014). Tough and flexible CNT–polymeric hybrid scaffolds for engineering cardiac constructs. Biomaterials.

[124] Liu Y., Lu J., Xu G., Wei J., Zhang Z., Li X. (2016). Tuning the conductivity and inner structure of electrospun fibers to promote cardiomyocyte elongation and synchronous beating. Mater. Sci. Eng. C.

[125] Martins A., Eng G., Caridade S., Mano J.F., Reis R.L., Vunjak-Novakovic G. (2014). Electrically Conductive Chitosan/Carbon Scaffolds for Cardiac Tissue Engineering. Biomacromolecules.

[126] Xu C., Police S., Rao N., Carpenter M.K. (2002). Characterization and Enrichment of Cardiomyocytes Derived From Human Embryonic Stem Cells. Circ. Res..

[127] Fu S., Ping P., Wang F., Luo L. (2018). Synthesis, secretion, function, metabolism and application of natriuretic peptides in heart failure. J. Biol. Eng..

[128] Talebi A., Labbaf S., Karimzadeh F., Masaeli E., Esfahani M.-H.N. (2020). Electroconductive Graphene-Containing Polymeric Patch: A Promising Platform for Future Cardiac Repair. ACS Biomater. Sci. Eng..

[129] Smith A.S.T., Yoo H., Yi H., Ahn E.H., Lee J.H., Shao G., Nagornyak E., Laflamme M., Murry C.E., Kim D.-H. (2017). Micro- and nano-patterned conductive graphene–PEG hybrid scaffolds for cardiac tissue engineering. Chem. Commun..

[130] Shin S.R., Zihlmann C., Akbari M., Assawes P., Cheung L., Zhang K., Manoharan V., Zhang Y.S., Yüksekkaya M., Wan K. (2016). Reduced Graphene Oxide-GelMA Hybrid Hydrogels as Scaffolds for Cardiac Tissue Engineering. Small.

[131] Shevach M., Fleischer S., Shapira A., Dvir T. (2014). Gold Nanoparticle-Decellularized Matrix Hybrids for Cardiac Tissue Engineering. Nano Lett..

[132] Peña B., Maldonado M., Bonham A.J., Aguado B.A., Dominguez-Alfaro A., Laughter M., Rowland T.J., Bardill J., Farnsworth N.L., Ramon N.A. (2019). Gold Nanoparticle-Functionalized Reverse Thermal Gel for Tissue Engineering Applications. ACS Appl. Mater. Interfaces.

[133] Allison S., Ahumada M., Andronic C., McNeill B., Variola F., Griffith M., Ruel M., Hamel V., Liang W., Suuronen E.J. (2017). Electroconductive nanoengineered biomimetic hybrid fibers for cardiac tissue engineering. J. Mater. Chem. B.

[134] Somasuntharam I., Yehl K., Carroll S.L., Maxwell J.T., Martinez M.D., Che P.-L., Brown M.E., Salaita K., Davis M.E. (2016). Knockdown of TNF-α by DNAzyme gold nanoparticles as an anti-inflammatory therapy for myocardial infarction. Biomaterials.

[135] Jia C., Chen H., Wei M., Chen X., Zhang Y., Cao L., Yuan P., Wang F., Yang G., Ma J. (2017). Gold nanoparticle-based miR155 antagonist macrophage delivery restores the cardiac function in ovariectomized diabetic mouse model. Int. J. Nanomed..

[136] Zeng Y., Du W.W., Wu Y., Yang Z., Awan F.M., Li X., Yang W., Zhang C., Yang Q., Yee A.J. (2017). A Circular RNA Binds To and Activates AKT Phosphorylation and Nuclear Localization Reducing Apoptosis and Enhancing Cardiac Repair. Theranostics.

[137] Li Y., Yu H., Zhao L., Zhu Y., Bai R., Jin Z., Fu Z., Zhang X., Su J., Liu H. (2020). Effects of carbon nanotube-mediated Caspase3 gene silencing on cardiomyocyte apoptosis and cardiac function during early acute myocardial infarction. Nanoscale.

[138] Paul A., Hasan A., Al Kindi H., Gaharwar A., Rao V.T.S., Nikkhah M., Shin S.R., Krafft D., Dokmeci M.R., Shum-Tim D. (2014). Injectable Graphene Oxide/Hydrogel-Based Angiogenic Gene Delivery System for Vasculogenesis and Cardiac Repair. ACS Nano.

[139] Yuan Z., Qin Q., Yuan M., Wang H., Li R. (2020). Development and novel design of clustery graphene oxide formed Conductive Silk hydrogel cell vesicle to repair and routine care of myocardial infarction: Investigation of its biological activity for cell delivery applications. J. Drug Deliv. Sci. Technol..

[140] Bao R., Tan B., Liang S., Zhang N., Wang W., Liu W. (2017). A π-π conjugation-containing soft and conductive injectable polymer hydrogel highly efficiently rebuilds cardiac function after myocardial infarction. Biomaterials.

[141] Zhou J., Chen J., Sun H., Qiu X., Mou Y., Liu Z., Zhao Y., Li X., Han Y., Duan C. (2015). Engineering the heart: Evaluation of conductive nanomaterials for improving implant integration and cardiac function. Sci. Rep..

[142] *Advancing Adverse Outcome Pathway (AOP) Development for Nanomaterial Risk Assessment and Categorisation Part 2: Case Study on Tissue Injury;* Safety of Manufactured Nanomaterials. https://www.oecd.org/officialdocuments/publicdisplaydocumentpdf/?cote=env/jm/%20mono(2020)34&doclanguage=en.

[143] *Ability of Biopersistent/Biodurable Manufactured Nanomaterials (MNs) to Induce Lysosomal Membrane Permeabilization (LMP) as a Prediction of Their Long-Term Toxic Effects;* Safety of Manufactured Nanomaterials. https://www.oecd.org/env/ehs/nanosafety/publications-series-safety-manufactured-nanomaterials.htm.

[144] Oomen A.G., Steinhäuser K.G., Bleeker E.A., van Broekhuizen F., Sips A., Dekkers S., Wijnhoven S.W., Sayre P.G. (2017). Risk assessment frameworks for nanomaterials: Scope, link to regulations, applicability, and outline for future directions in view of needed increase in efficiency. NanoImpact.

[145] Lamon L., Aschberger K., Asturiol D., Richarz A., Worth A. (2019). Grouping of nanomaterials to read-across hazard endpoints: A review. Nanotoxicology.

[146] Arts J.H.E., Hadi M., Irfan M.-A., Keene A.M., Kreiling R., Lyon D., Maier M., Michel K., Petry T., Sauer U.G. (2015). A decision-making framework for the grouping and testing of nanomaterials (DF4nanoGrouping). Regul. Toxicol. Pharmacol..

[147] Friedman E., McMahon M. TO VAD OR NOT TO VAD: That is the question. Improving the experience of receiving a Ventricular Assist Device (VAD). Proceedings of the International Symposium on Human Factors and Ergonomics in Health Care.

[148] Moayedi Y., Fan C.P.S., Cherikh W.S., Stehlik J., Teuteberg J.J., Ross H.J., Khush K.K. (2019). Survival Outcomes After Heart Transplantation: Does Recipient Sex Matter?. Circ. Heart Fail..

[149] Sommer G., Schriefl A.J., Andrä M., Sacherer M., Viertler C., Wolinski H., Holzapfel G.A. (2015). Biomechanical properties and microstructure of human ventricular myocardium. Acta Biomater..

